# The *Campylobacter jejuni* CiaD effector protein activates MAP kinase signaling pathways and is required for the development of disease

**DOI:** 10.1186/1478-811X-11-79

**Published:** 2013-10-21

**Authors:** Derrick R Samuelson, Tyson P Eucker, Julia A Bell, Leslie Dybas, Linda S Mansfield, Michael E Konkel

**Affiliations:** 1 School of Molecular Biosciences, Washington State University, College of Veterinary Medicine, Life Sciences Bldg. Room 302c, Pullman, WA 99164-7520, USA; 2 Comparative Enteric Diseases Laboratory, Michigan State University, College of Microbiology & Molecular Genetics, East Lansing, MI 48824, USA

**Keywords:** Invasion, Erk 1/2, p38, IL-8 secretion, Type III secretion system

## Abstract

**Background:**

Enteric pathogens utilize a distinct set of proteins to modulate host cell signaling events that promote host cell invasion, induction of the inflammatory response, and intracellular survival. Human infection with *Campylobacter jejuni*, the causative agent of campylobacteriosis, is characterized by diarrhea containing blood and leukocytes. The clinical presentation of acute disease, which is consistent with cellular invasion, requires the delivery of the *Campylobacter* invasion antigens (Cia) to the cytosol of host cells via a flagellar Type III Secretion System (T3SS). We identified a novel T3SS effector protein, which we termed CiaD that is exported from the *C*. *jejuni* flagellum and delivered to the cytosol of host cells.

**Results:**

We show that the host cell kinases p38 and Erk 1/2 are activated by CiaD, resulting in the secretion of interleukin-8 (IL-8) from host cells. Additional experiments revealed that CiaD-mediated activation of p38 and Erk 1/2 are required for maximal invasion of host cells by *C*. *jejuni*. CiaD contributes to disease, as evidenced by infection of IL-10 knockout mice. Noteworthy is that CiaD contains a Mitogen-activated protein (MAP) kinase-docking site that is found within effector proteins produced by other enteric pathogens. These findings indicate that *C*. *jejuni* activates the MAP kinase signaling pathways Erk 1/2 and p38 to promote cellular invasion and the release of the IL-8 pro-inflammatory chemokine.

**Conclusions:**

The identification of a novel T3SS effector protein from *C*. *jejuni* significantly expands the knowledge of virulence proteins associated with *C*. *jejuni* pathogenesis and provides greater insight into the mechanism utilized by *C*. *jejuni* to invade host cells.

## Lay abstract

In contrast to the conventional view that the flagellum is only used for bacterial motility, *Campylobacter jejuni* also uses the flagellum as a Type III Secretion System (T3SS). A subset of proteins exported from the flagellum are delivered to the cytosol of host cell, where they modify host cell signaling events to the benefit of the bacterial pathogen. Here we report that *C*. *jejuni*, which is the leading bacterial cause of food-borne disease worldwide, possesses a novel T3SS virulence protein that we termed CiaD. We show that CiaD is required for the invasion of host cells and for the secretion of the inflammatory chemokine interleukin-8 from host cells. We also show that the newly identified virulence protein CiaD is required for the development of disease. The fact that CiaD is required of the development of disease provides a significant advancement in the understanding of *C*. *jejuni* pathogenesis.

## Background

*Campylobacter jejuni* is a leading cause of gastroenteritis worldwide, causing ~1.4 to 2.3 million cases each year in the United States [1,2]. A serious complication of *C*. *jejuni* infection is the development of Guillain–Barré syndrome (GBS), an autoimmune disease affecting the peripheral nervous system [3]. The ability of *C*. *jejuni* to cause acute disease is a complex multifactorial process, requiring cell adherence, invasion, and intracellular survival [4-10]. Key to host cell invasion and survival is the synthesis of the *Campylobacter* invasion antigens (Cia) [11]. Cia proteins are exported from the bacterium’s flagellar Type III Secretion System (T3SS) and are delivered to the host cell to promote maximal cell invasion [12].

Only three (CiaB, CiaC, and CiaI) of ~18 Cia proteins have been identified to date [4,13,14]. While the precise functions of these proteins are not known, *in vivo* studies suggest that they contribute to disease. More specifically, inoculation of piglets with a *C*. *jejuni* wild-type strain and *ciaB* mutant results in different clinical signs. Piglets infected with *C*. *jejuni* develop diarrhea within 24 hours, and exhibit severe histological lesions, including shortening of the villi and production of an exudate in the lumen. In contrast, piglets inoculated with a *C*. *jejuni ciaB*-knockout mutant do not develop diarrhea until 3 days post-infection and exhibit only minor histological lesions [14]. Introducing a wild-type copy of the *ciaB* gene into the *C*. *jejuni ciaB* mutant restored the isolate’s virulence [15]. Additional work is needed to identify and characterize the Cia proteins to gain a better understanding of *C*. *jejuni* pathogenesis.

*C*. *jejuni* invades the cells lining the gastrointestinal tract and induces a potent inflammatory response characterized by the secretion of interleukin-8 (IL-8). However, little is known about the mechanism underlying the induction of IL-8 secretion by *C*. *jejuni*. Bacterial factors, such as the flagellum and CpG dinucleotide, are typical immune stimulators [16]. However, Toll-like receptor 5 (TLR5) is not stimulated by the *C*. *jejuni* flagellum [17,18]. Similarly, TLR9, which recognizes CpG dinucleotides, is not efficiently stimulated by *C*. *jejuni*[16]. While *C*. *jejuni* is clearly stimulating a proinflammatory response, the mechanism of immune activation is not completely understood.

Researchers have found that the NF-κB pathway is activated by *C*. *jejuni*. The NF-κB pathway is activated by JlpA, CdtABC, and peptidoglycan (intracellular NOD-1 receptors) [19-21]. While both the NF-κB and AP-1 transcriptional activators are required for expression of the gene encoding IL-8, also known as huCXCL8 [22], the mechanism of activation of AP-1 by *C*. *jejuni* is not known. In addition to their role in cell growth and differentiation, Erk 1/2 and p38 serve as important activators of the immune response in non-phagocytic cells through the activation of AP-1. Several labs have reported that Erk 1/2 and p38 signaling pathways are activated by *C*. *jejuni*, and that the activation of these pathways is dependent on bacterial *de novo* protein synthesis and a functional flagellum [18,23,24]. The *C*. *jejuni* factors necessary for Erk 1/2 and p38 mediated IL-8 secretion are not known.

We hypothesized that *C*. *jejuni* delivers one or more of the Cia proteins to host cells where they trigger the induction of IL-8 secretion from host cells. Here we identify a novel protein (Cj0788), which we termed *Campylobacter* invasion antigen D (CiaD), that is secreted via the flagellar T3SS. CiaD is required for maximal *C*. *jejuni* invasion and IL-8 secretion from human INT 407 epithelial cells. We also show that CiaD is required for the development of acute disease *in vivo*. Specifically, the *C*. *jejuni* wild-type strain resulted in disease characterized by a thickening of the gastrointestinal tract wall, enlarged ileocecocolic lymph nodes, and bloody lumen contents in cecum and colon, which was absent in mice infected with the *C*. *jejuni ciaD* mutant. These data are significant, as this is the first time that a *C*. *jejuni* effector protein has been shown to contribute to the development of disease in a mouse model.

## Results

### The flagellum is required for CiaD delivery to host epithelial cells

**Figure 1:**
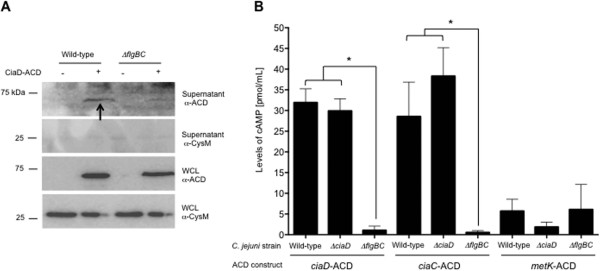
**CiaD is delivered to the cytosol of human epithelial cells. (A)** CiaD is secreted from *C*. *jejuni*. A *C*. *jejuni* wild-type strain transformed with the pRY111 vector harboring CiaD fused to the adenylate cyclase domain (CiaD-ACD, + designation) was analyzed by immunoblot analysis. CysM is a cytoplasmic protein (*O*-acetylserine sulfhydrylase B) involved in cysteine biosynthesis (*M*_r_ = 32.4 kDa). Also included was a C. *jejuni* wild-type strain without the pRY111 shuttle vector as a negative control (- designation) and a *C*. *jejuni flgBC* mutant expressing CiaD-ACD and a pRY111 shuttle vector. **(B)** CiaD is delivered to host cells. A *C*. *jejuni* wild-type strain, *ciaD* mutant, and *flgBC* mutant were transformed with pRY111 vector harboring the *ciaD*-ACD, *ciaC*-ACD, and *metK*-ACD inserts. *C*. *jejuni* harboring the *metK*-ACD vector was used as a negative control. The CiaC-ACD, a known delivered protein, was included as a positive control. The delivery of the CiaD-ACD, CiaC-ACD, and MetK-ACD fusion proteins to the cytosol of human INT 407 cells was determined using the ACD delivery assay described in Methods. The asterisk indicates that the amount of cAMP produced in the wild-type strain was significantly greater than the value obtained from the *flgBC* mutant, as judged by one-way ANOVA followed by post-hoc Tukey’s analysis (*P* < 0.05). Each error bar represents ± the standard error of the mean (SEM) of N = 9 samples from 3 independent experiments.

Previous work in our lab led to the identification of 42 proteins that contain a putative *C*. *jejuni* flagellar T3SS export signal [4]. We sought to determine if one of these proteins, Cj0788, designated *Campylobacter* invasion antigen D (CiaD), is secreted by *C*. *jejuni*. We tested if CiaD is secreted from a *C*. *jejuni* wild-type strain and *ciaD* mutant harboring a plasmid encoding CiaD fused to the adenylate cyclase domain (ACD) of the CyaA protein from *Bordetella pertussis*. The CiaD-ACD fusion protein was secreted from the *C*. *jejuni* wild-type strain and a *ciaD* mutant but not the *flgBC* flagellar mutant, as judged by immunoblot analysis using an ACD specific antibody (Figure 1A and Additional file 1: Figure S1A). To determine if CiaD is required for Cia secretion, we tested if a second Cia protein (CiaC) could be exported from the *ciaD* mutant transformed with a construct harboring CiaC-ACD. In addition, the *ciaD* mutant was transformed with a construct harboring MetK-ACD (a known cytosolic protein), as a negative control. In contrast to MetK, the CiaC effector protein was secreted from the *ciaD* mutant (Additional file 1: Figure S1A). These assays show that CiaD is secreted from a *C*. *jejuni* wild-type strain and is not required for the secretion of other Cia proteins.

To determine if CiaD is delivered to the cytosol of human epithelial cells, INT 407 human intestinal cells were infected with *C*. *jejuni* transformed with the CiaD-ACD construct and host cell cAMP levels were measured via ELISA as described previously [12]. The *C*. *jejuni* wild-type strain transformed with the CiaC-ACD and MetK-ACD constructs were included as positive and negative controls, respectively. All of the fusion proteins were synthesized in the transformed *C. jejuni* isolates (Additional file 1: Figure S1B). However, in comparison to the negative control, a significant increase (P < 0.05) in the level of cAMP was observed in cells inoculated with the *C*. *jejuni* CiaD-ACD strain (Figure 1B). The delivery of CiaD was found to be dependent on a functional flagellum, as infection of INT 407 cells with a *C*. *jejuni flgBC* mutant (secretion negative control) transformed with the CiaD-ACD construct resulted in a significant decrease in cAMP detected as compared to the *C*. *jejuni* wild-type strain (Figure 1B). We utilized the Cia delivery assay to determine if the *ciaD* mutant could deliver CiaC to a host cell. Again, in contrast to MetK, the CiaC effector protein was delivered from the *ciaD* mutant to host cells, as judged by a significant increase in cAMP versus the controls.

### CiaD induces the secretion of IL-8 from epithelial cells

**Figure 2:**
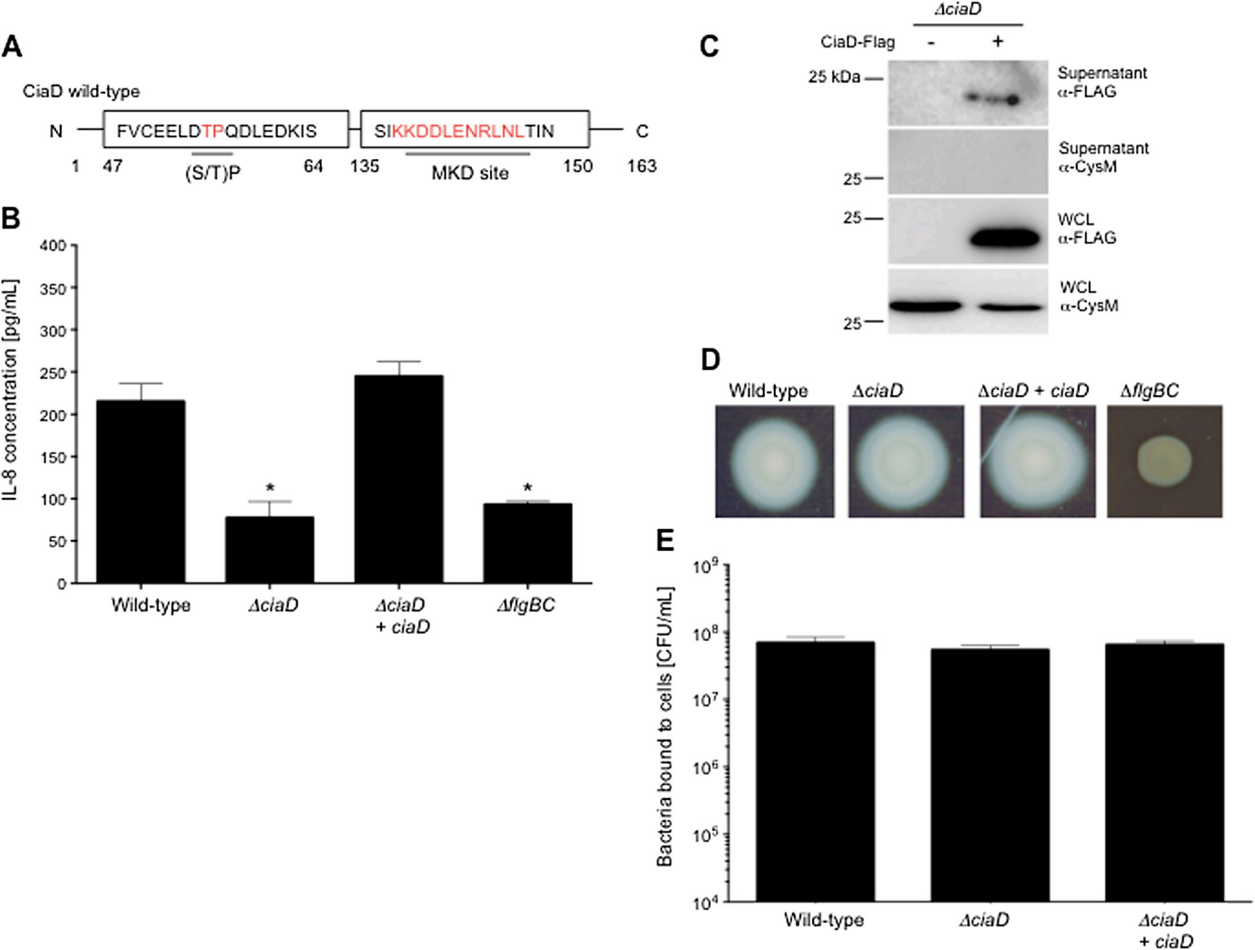
**CiaD is required for the maximal secretion of interleukin**-**8 from host cells. A)** Schematic representation of the CiaD Mitogen-activated protein kinase docking (MKD) motif residues 137–147 and the proline-directed phosphorylation site ((S/T)P) motif residue 54. **(B)** CiaD is required for the maximal secretion of IL-8 by INT 407 cells. The *flgBC* flagellar mutant was included as a negative control. The values obtained from uninfected cells were subtracted from all other values. The asterisks indicate a significant reduction in the amount of IL-8 detected in the supernatants compared to the value obtained using the *C*. *jejuni* wild-type strain, using a one-way ANOVA followed by post-hoc Tukey’s analysis (*P* < 0.05). Error bars represent ± SEM of N = 12 samples from 3 independent experiments. **(C)** CiaD is secreted from a *C*. *jejuni ciaD* mutant transformed with pRY111 vector harboring the CiaD-FLAG-tagged protein. Blots were probed with FLAG and CysM antibodies. Also included was a *C*. *jejuni ciaD* mutant without the pRY111 shuttle vector (-) as a negative control. **(D)** The *C*. *jejuni ciaD* mutant is motile. Motility assays were performed as outlined in Methods. **(E)** Deletion of *ciaD* does not have an effect on *C*. *jejuni* binding to host cells at 30 min post-infection.

An effector protein is defined as a protein delivered from a pathogen to a host cell that ultimately functions to alter host cell behavior. Based on the finding that CiaD is delivered to host cells, we postulated that CiaD acts as an effector protein. *In*-*silico* analysis was used to look for eukaryotic domains in CiaD. The web-based program Eukaryotic Linear Motif (ELM) revealed that CiaD contained a Mitogen-activated protein kinase docking (MKD) motif (probability score = 0.0043) and a proline-directed phosphorylation ((S/T)P) motif (probability score = 0.0154) (Figure 2A) [25,26]. In addition, the Phyre^2^ protein folding prediction software [27] revealed that CiaD contains a putative nucleotidyltransferase folding domain (52.6% confidence) (not shown). Nucleotidyltransferase domains are commonly found in bacterial effector proteins involved in adenylylation of RhoGTPase leading to actin remodeling [28]. The presence of these eukaryotic domains raised the possibility that CiaD might alter host cell behavior.

Investigators have reported that *C*. *jejuni de novo* protein synthesis is required for maximal secretion of IL-8 from host cells [18,29]. Consistent with these reports, we found that incubation of *C*. *jejuni* with chloramphenicol (a protein synthesis inhibitor) for 30 min prior to inoculation of human INT 407 cells reduced the amount of IL-8 secreted from the host cells as well as *C*. *jejuni* invasion (Additional file 2: Figure S2A and S2B). Noteworthy is that INT 407 cells are responsive to innate immune signaling molecules that engage TLR4 and TLR2 [30]. Previous work has also indicated that the genes encoding the Cia proteins are induced when *C*. *jejuni* are cultured with epithelial cells [11]. Taken together, these findings raised the possibility that a Cia protein was required for IL-8 induction. Based on the presence of the MKD site in CiaD and the link between IL-8 induction and MAP kinase signaling, we measured the IL-8 pro-inflammatory chemokine in the supernatants collected from INT 407 cells inoculated with the *C*. *jejuni* wild-type strain and *ciaD* mutant. The *C*. *jejuni flgBC* mutant was included as a negative control; this mutant binds to the cells at levels similar to that of a *C*. *jejuni* wild-type strain but is deficient in Cia protein secretion and delivery (not shown). A significant decrease (P < 0.01) in secreted IL-8 was observed in cells inoculated with the *C*. *jejuni ciaD* and *flgBC* mutants when compared to a *C*. *jejuni* wild-type strain (Figure 2B). Additionally, IL-8 secretion from host cells was not altered by removal of non-adherent bacteria by washing or in the presence of non-adherent bacteria (not shown).

Several additional findings indicate that CiaD is, in part, responsible for inducing IL-8 secretion from host cells. First, insertion of a wild-type copy of the *ciaD* gene driven by the constitutive promoter *hupB* into the *ciaD* mutant *in trans* (complemented strain) resulted in an isolate that displayed an IL-8 secretion phenotype indistinguishable from that of the wild-type strain (Figure 2B). Second, ectopic expression of a gene that encodes CiaD-EGFP in host INT 407 cells resulted in a moderate increase in IL-8 secretion as compared to ectopic expression of EGFP only (Additional file 3: Figure S3). We further demonstrated that: a) CiaD protein is secreted from the *ciaD* complemented isolate, as judged by immunoblot analysis (Figure 2C); b) the *C*. *jejuni ciaD* mutant and *ciaD* complemented isolate are both motile, as judged by motility assays, indicating a functional flagellum (Figure 2D); and c) the *C*. *jejuni* wild-type strain, *ciaD* mutant, and *ciaD* complemented isolate bound to INT 407 cells with equal efficiency, as judged by a cell binding assay (Figure 2E). Moreover, no difference was observed in the binding of all of the isolates to the host cells over an eight-hour period (not shown). In separate assays, we found that the inoculation of INT 407 cells with an isolate that contained a knockout in Cj0789, which is the gene immediately downstream of *ciaD*, resulted in a similar level of secreted IL-8 as that of the wild-type strain (Additional file 4: Figure S4). Consistent with the proposal that the delivery of CiaD to host cells requires bacteria-host cell contact, the addition of supernatants containing the Cia proteins to INT 407 cells did not induce IL-8 secretion (not shown). Based on these data, we concluded that CiaD is an effector protein that is involved in the induction of IL-8 secretion.

### CiaD is required for cell invasion

**Figure 3:**
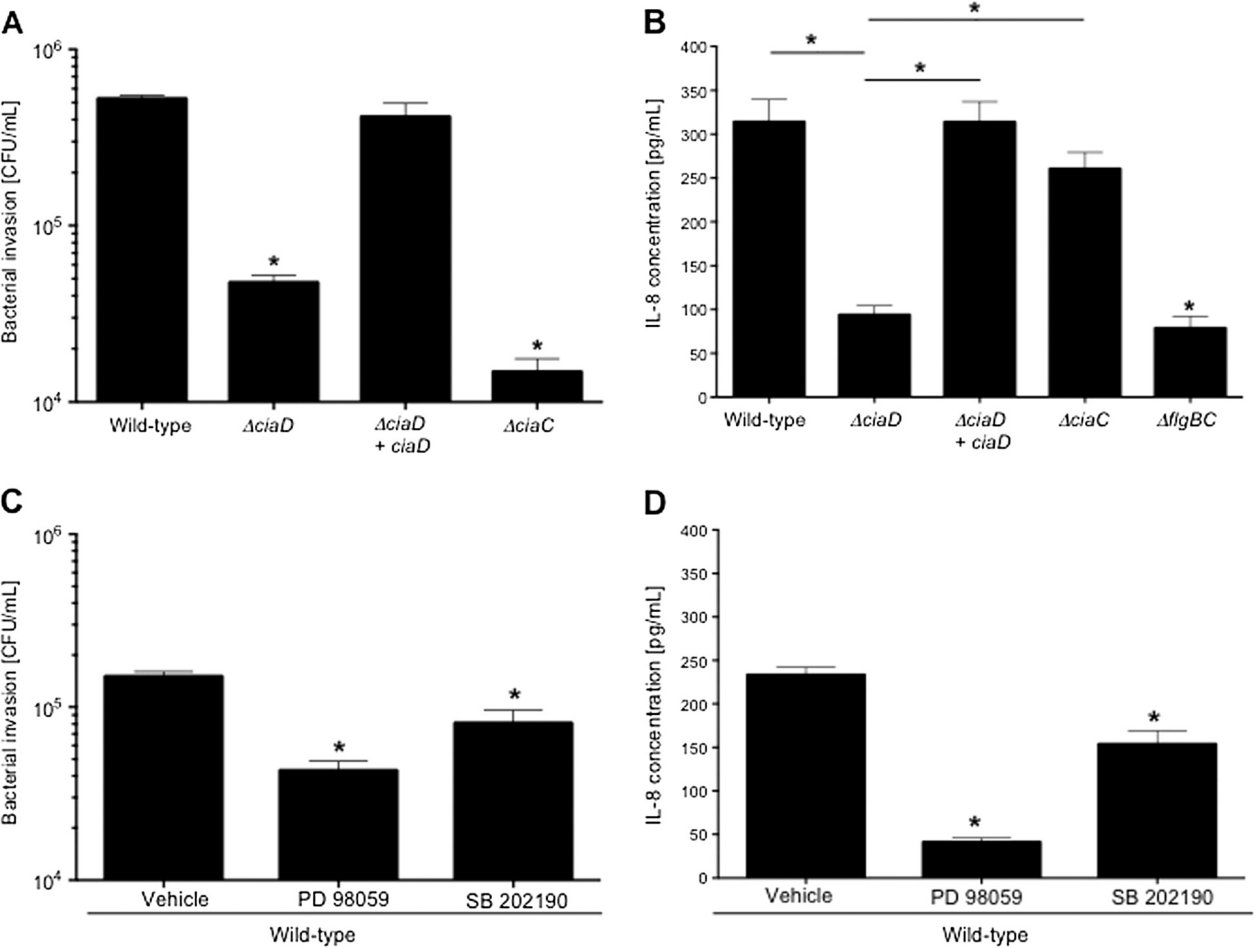
**IL**-**8 secretion from INT 407 cells is not invasion dependent**, **but MAPK activation is required for host cells invasion by *C.jejuni*. (A)** A *C*. *jejuni ciaD* mutant is deficient in maximal invasion of INT 407 cells. The *C*. *jejuni ciaC* mutant was included as an invasion deficient control. **(B)** IL-8 secretion does not require bacterial invasion. INT 407 cells were inoculated with various *C*. *jejuni* isolates, incubated for 24 hr, and IL-8 quantified using an IL-8 ELISA as described in Methods. The *C*. *jejuni flgBC* flagellar mutant (Cia secretion negative) and *C*. *jejuni ciaC* mutant (CiaC secretion negative and invasion deficient) were included as negative controls. **(C)** MAP kinase activation is required for *C*. *jejuni* invasion. Inhibitors to Erk 1/2 (PD98059) and p38 (SB 202190) were added to INT 407 cells for 30 min prior to the addition of a *C*. *jejuni* wild-type strain. **(D)** MAP kinase activation is required for IL-8 secretion. Inhibitors of Erk 1/2 and p38 were added to INT 407 cells for 30 min prior to inoculation with a *C*. *jejuni* wild-type strain. The values obtained from uninfected cells were subtracted from all other values. The asterisks indicate a significant reduction compared to the value obtained for the *C*. *jejuni* wild-type strain, according to one-way ANOVA followed by post-hoc Tukey’s analysis (*P* < 0.05). Error bars represent ± SEM of N = 12 samples from 3 independent experiments.

We performed experiments to determine if there is a possible link between IL-8 induction and *C*. *jejuni* invasion of host cells. The ability of the *C*. *jejuni ciaD* mutant to invade INT 407 cells was determined using the gentamicin-protection assay. The *C*. *jejuni ciaC* mutant was included as a control, as this mutant displays a significant reduction in cell invasion compared to a wild-type strain of *C*. *jejuni*. Both the *C*. *jejuni ciaD* and *ciaC* mutants exhibited a reduction in cell invasion when compared to a wild-type strain (Figure 3A). To determine if cell invasion is required to induce IL-8 secretion from a host cell, INT 407 cells were inoculated with the *C*. *jejuni ciaD* and *ciaC* mutants and the amount of IL-8 secreted into the supernatants was determined. Consistent with our previous findings, CiaD was required to induce maximal IL-8 secretion (Figure 2B). We also found that the *ciaC* mutant induced levels of IL-8 secretion indistinguishable from the *C*. *jejuni* wild-type strain (Figure 3B). This finding suggested that invasion and IL-8 secretion are not directly linked.

To address the role of MAP kinase signaling in *C*. *jejuni* induction of IL-8 secretion and host cell invasion, assays were performed in the presence of cellular inhibitors to Erk 1/2 (PD98059) and p38 (SB202190). Inhibition of Erk 1/2 and p38 resulted in a significant reduction (P < 0.01) in the number of *C*. *jejuni* internalized (Figure 3C) and the amount of secreted IL-8 (Figure 3D). Consistent with these findings, we found that the amount of IL-8 secreted by the host cells inoculated with the CiaD mutant was reduced significantly when the activation of Erk 1/2 and p38 were inhibited (Additional file 5: Figure S5). Specifically, inhibition of Erk 1/2 results in a 70% reduction in the amount of IL-8 secreted from host cells infected with a *C*. *jejuni* wild-type strain, similarly inhibition of Erk 1/2 resulted also in a reduction in IL-8 secreted from host cells that were infected with the *C*. *jejuni ciaD* mutant. These results are consistent with the fact that the *C*. *jejuni ciaD* mutant activates Erk 1/2 to a level that is slightly above that of cells only. Moreover, the addition of exogenous IL-8 to Caco-2 cells, an intestinal cell line that is responsive to IL-8 because of the presence of the CXCR1 and CXCR2 receptors, did not restore the invasiveness of the *C*. *jejuni ciaD* mutant to that of a *C*. *jejuni* wild-type strain (Additional file 6: Figure S6). This finding suggests that the invasion phenotype of the *ciaD* mutant is due to a lack in the initiation of cellular signaling events specific to invasion, and not from the failure to induce the secretion of IL-8 from host cells. We also confirmed that Caco-2 cells are responsive to IL-8, using immunoblot analysis to quantify phospho-Akt; Akt is a downstream target of the CXCR1/2 receptors and is activated by IL-8 [31] (Additional file 6: Figure S6B). Together, these experiments revealed that *C*. *jejuni* must activate components of the MAP kinase signaling pathway for both cellular invasion and the secretion of IL-8, and that CiaD contributes to this activation.

### CiaD activates of the MAP kinase signaling pathway

**Figure 4:**
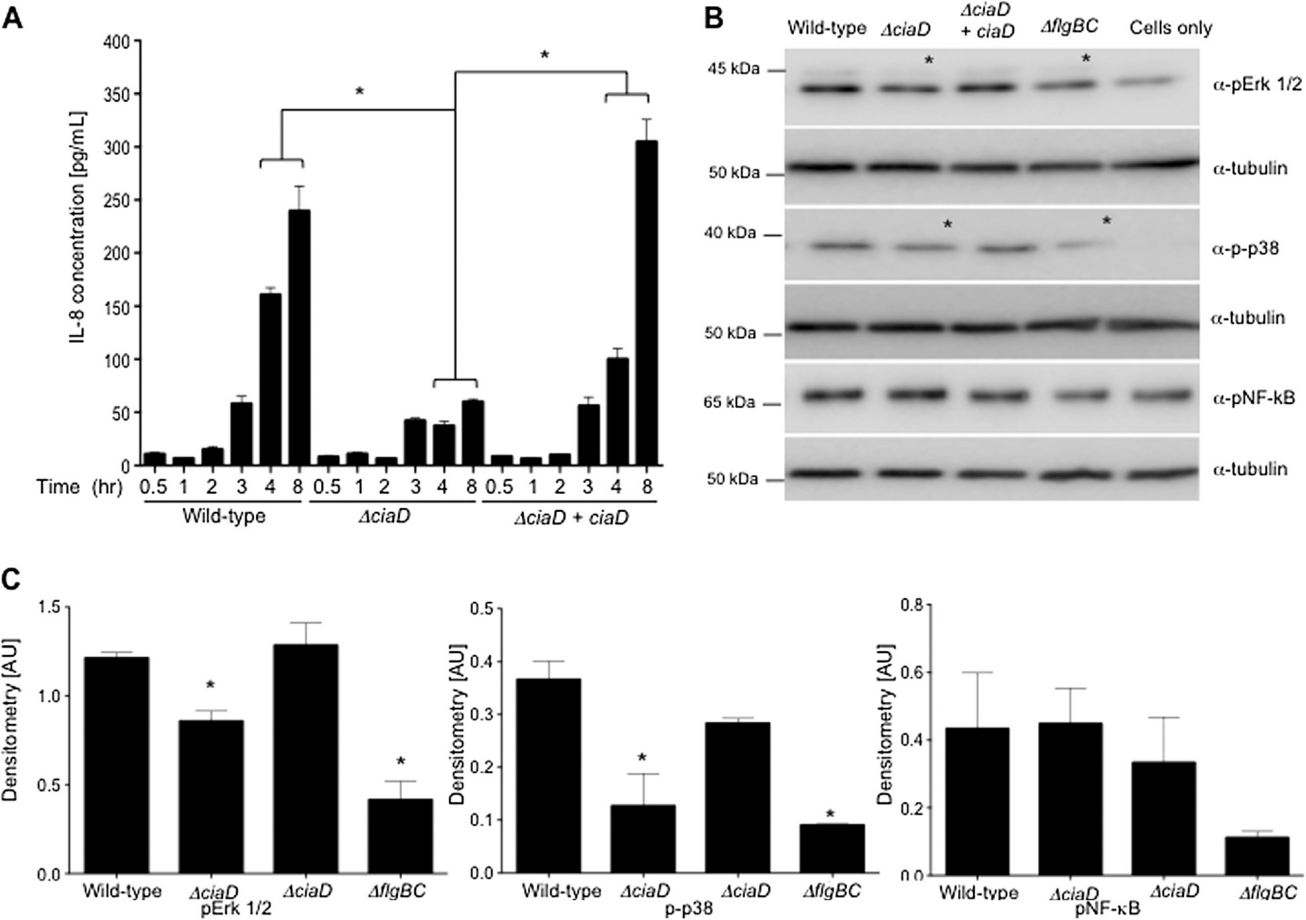
**CiaD is required for activation of the MAP kinase signaling cascade. (A)** The amount of IL-8 in supernatants is significantly increased 4 hr post infection in the *C*. *jejuni* wild-type strain when compared to the *ciaD* mutant. **(B)** Maximal activation of p38 and Erk 1/2 requires CiaD, as judged by immunoblots. Immunoblots were probed with phospho-specific antibodies to Erk 1/2 (*M*_r_ = 42 and 44 kDa), p38 (*M*_r_ = 38 kDa), and NF-κB p65 subunit phospho-S536 (*M*_r_ = 65 kDa). All blots were stripped and re-probed with an anti-tubulin (*M*_r_ = 52 kDa) antibody. **(C)** Maximal activation of p38 and Erk 1/2 requires CiaD. Densitometry was performed on the immunoblots shown in Panel B. The value obtained for cells only was subtracted from all other values. The asterisks indicate a significant difference in values compared to the controls, as judged by one-way ANOVA followed by post-hoc Tukey’s analysis (*P* < 0.05). Error bars represent ± SEM of N = 6 samples from 6 independent experiments.

Based on the presence of the Mitogen-activated protein kinase docking (MKD) motif in CiaD, experiments were performed to determine if *C*. *jejuni* activated the MAP kinase signaling pathway in a CiaD dependent manner. Prior to performing these experiments, we determined that the *C*. *jejuni* wild-type strain induces significantly more IL-8 than the *ciaD* mutant at 4 hr post-inoculation of INT 407 cells (Figure 4A). Based on the kinetics of IL-8 secretion, we performed experiments to identify the cellular signaling pathways that are activated by *C*. *jejuni* at 3 hr post inoculation. The use of a MAP kinase phosphor-array screen revealed that the *C*. *jejuni ciaD* mutant did not activate Erk 1/2 and p38 to the same extent as inoculation of the INT 407 cells with the wild-type strain (Additional file 7: Figure S7). These results were confirmed by immunoblot analysis (Figure 4B and Figure 4C). The partial activation (above cells only) of Erk 1/2 by the *ciaD* mutant is consistent with the fact that Erk 1/2 is also partially activated in response to host cell binding mediated by FlpA [32]. In contrast to the *C*. *jejuni ciaD* and *flgBC* (Cia protein secretion-negative) mutants, the *C*. *jejuni* wild-type strain and *ciaD* mutant harboring a wild-type copy of the *ciaD* gene (complemented *ciaD* isolate) resulted in the activation of Erk 1/2 and p38 as judged by an increase in band intensity of the phosphorylated form of the protein. Even though the *C*. *jejuni* wild-type strain, *ciaD* mutant, and complemented *ciaD* isolate resulted in greater activation of NF-κB (p65 subunit phospho-S536) compared to the *flgBC* mutant, significant differences were not observed in the amount of NF-κB activation between the isolates (Figure 4B and 4C). This finding suggested that a protein or other bacterial component other than CiaD is responsible for the activation of NF-κB. Taken together, our results indicate that CiaD participates in the activation of the MAP kinase signaling molecules Erk 1/2 and p38.

### The MAP Kinase docking motif of CiaD is required for IL-8 secretion and host cell invasion

**Figure 5:**
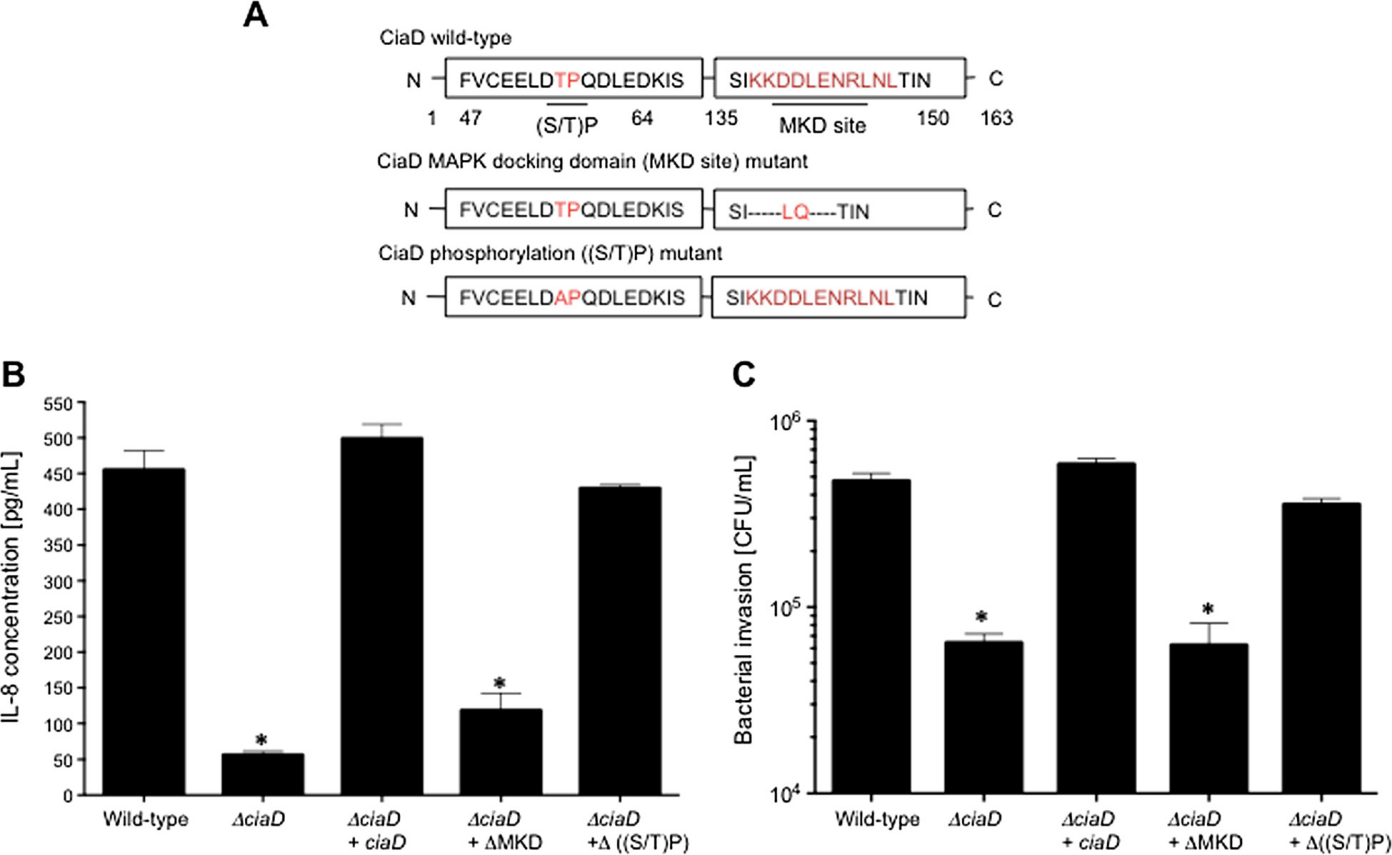
**The MKD site of CiaD is necessary for cellular invasion and IL**-**8 secretion. (A)** Schematic representation of the CiaD wild-type protein, the CiaD MAP kinase docking motif (ΔMKD site) mutant protein, and CiaD proline directed-phosphorylation site (Δ(S/T)P) mutant protein. **(B)** The MKD site of CiaD is required for *C*. *jejuni* to induce maximal IL-8 secretion from INT 407 cells. The value obtained from cells only was subtracted from all other values. **(C)** The MKD site of CiaD is required for maximal *C*. *jejuni* invasion of INT 407 cells. The asterisks indicate a significant difference compared to the *C*. *jejuni* wild-type strain, as judged by one-way ANOVA followed by post-hoc Tukey’s analysis (*P* < 0.05). Error bars represent ± SEM of N = 12 samples from 3 independent experiments.

Mutational analysis was used to determine whether the putative eukaryotic domains of CiaD are functional. Two *C*. *jejuni ciaD* mutants were generated; the MAP kinase docking motif was deleted (ΔMKD) in one mutant and the proline-directed phosphorylation motif was changed to an alanine (ΔS/T)P) in the other mutant (Figure 5A). Immunoblot analysis revealed that both CiaD recombinant proteins were synthesized in the *ciaD* mutant (Additional file 8: Figure S8A). Importantly, all of the isolates were motile (Additional file 8: Figure S8B). Experiments were then performed to evaluate the ability of the CiaD ΔMKD mutant and CiaD (ΔS/T)P mutant to induce IL-8 secretion and to invade human INT 407 epithelial cells. The CiaD ΔMKD mutant was unable to induce secretion of IL-8 from host cells, and was equally impaired in its ability to invade host cells (Figure 5B and C). In contrast, the CiaD (ΔS/T)P mutant induced the secretion of IL-8 and invaded host cells to a level that was indistinguishable from the *C*. *jejuni* wild-type strain (Figure 5B and C). These results indicate that the MKD motif of CiaD is required to modify a host cell signaling pathway(s), and that the interaction of CiaD with a host protein leads to IL-8 secretion and cell invasion.

### The phenotype of the *ciaD* mutant is the same in *C*. *jejuni* F38011 and 11168 strains

**Figure 6:**
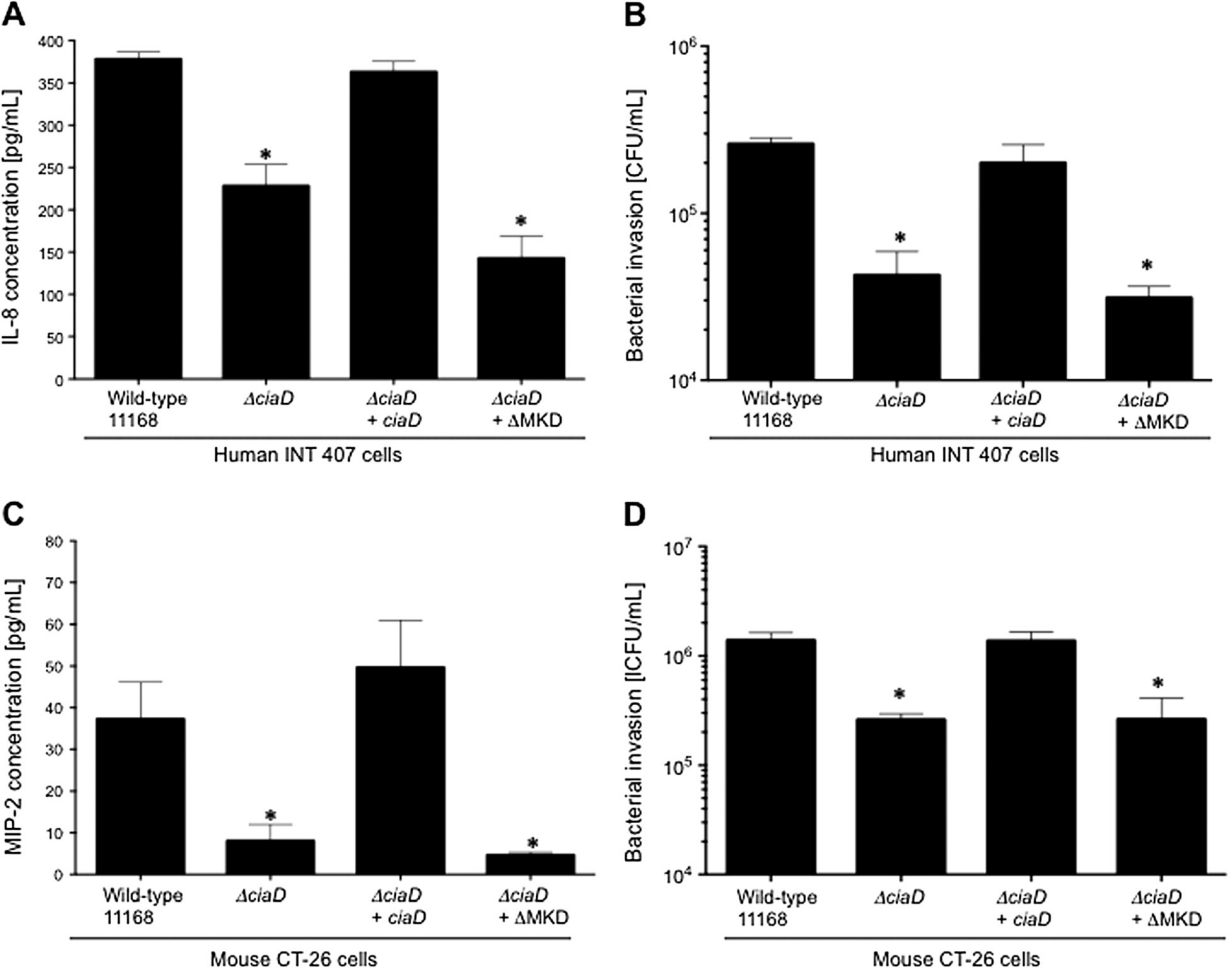
**CiaD function is conserved between strains of *C***. ***jejuni *and different cell types. (A)** The MAP kinase docking motif of CiaD is required for IL-8 secretion from human INT 407 cells. Cells only were subtracted from all shown values. **(B)** The MAP kinase docking motif of CiaD is required for maximal invasion of human INT 407 cells. **(C)** The MAP kinase docking motif of CiaD is required for MIP-2 secretion from murine CT-26 cells. MIP-2 concentrations were quantified using an MIP-2 ELISA as described in Methods. Cells only were subtracted from all shown values. **(D)** The MAP kinase docking motif of CiaD is required for maximal invasion of murine CT-26 cells. The asterisks indicate a significant difference in the value versus that of the *C*. *jejuni* wild-type strain, as judged by one-way ANOVA followed by post-hoc Tukey’s analysis (*P* < 0.05). Error bars represent ± SEM of N = 12 samples from 3 independent experiments.

Prior to assessing whether *ciaD* contributes to the development of acute disease *in vivo*, it was first necessary to regenerate the *ciaD* mutant in the *C*. *jejuni* 11168 mouse adapted strain and confirm if the mutants behaved in a similar fashion as the *C*. *jejuni* F38011 *ciaD* mutant. More specifically, four isolates were used in these experiments: a) the *C*. *jejuni* 11168 wild-type strain; b) the *C*. *jejuni* 11168 *ciaD* mutant; c) the *ciaD* mutant that synthesizes a CiaD wild-type protein; and d) the *ciaD* mutant that synthesizes the CiaD ΔMKD recombinant protein. We did not regenerate a *ciaD* mutant that synthesizes the CiaD Δ(S/T)P mutant protein, as the *C*. *jejuni* F38011 isolate that synthesizes this recombinant protein did not yield a unique phenotype. The CiaD protein was readily detected in the *C*. *jejuni* 11168 *ciaD* mutant transformed with the vectors that encode for the CiaD wild-type protein and CiaD ΔMKD-site protein (Additional file 8: Figure S8A). All of the isolates tested were motile (Additional file 8: Figure S8B). We then measured the amount of IL-8 and MIP-2 (a homologue of IL-8 in mice) secreted from human INT 407 cells and mouse CT-26 cells inoculated with the various *C*. *jejuni* strains, respectively. The results obtained with the *C*. *jejuni* 11168 isolates in INT 407 cells mirrored those obtained with the *C*. *jejuni* F38011 strain in INT 407 cells for IL-8 secretion and cell invasion (Figure 6A and B). Similarly, we observed that the *ciaD* mutant and *ciaD* mutant that synthesize the CiaD ΔMKD-site recombinant protein were deficient in the ability to induce MIP-2 secretion and invade CT-26 cells (Figure 6C and D). These data indicate that the phenotypes of the 11168 mouse adapted isolates are indistinguishable to that of the *C*. *jejuni* F38011 isolates, and that CiaD is required for MIP-2 secretion and cell invasion. Given these findings, we then performed *in vivo* experiments to determine the contribution of CiaD to campylobacteriosis.

### CiaD is required for the development of disease

**Figure 7:**
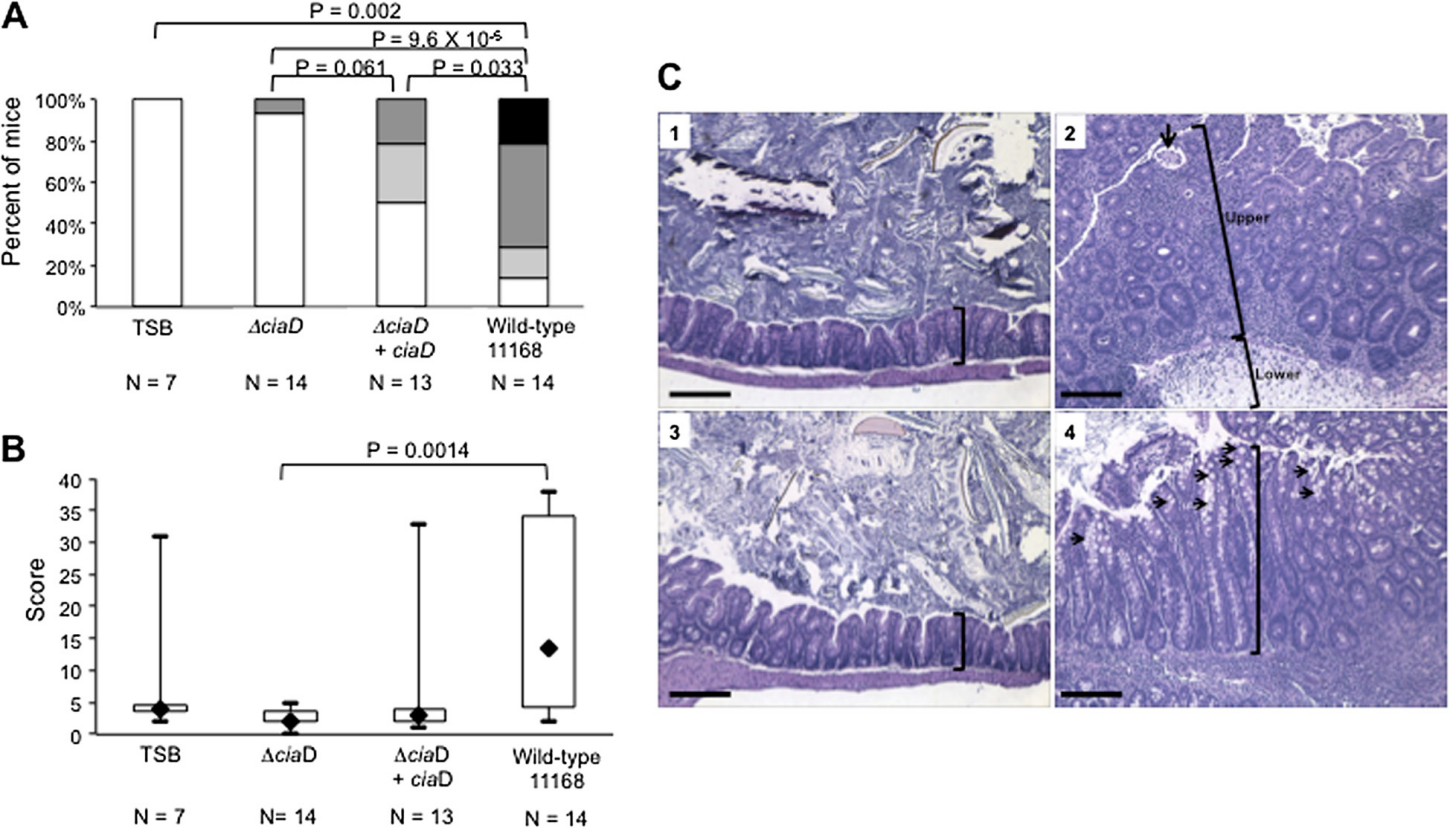
**CiaD is required for the development of severe disease. (A)** Gross pathology of IL-10^-/-^ mice infected with *C*. *jejuni*. Significant differences in gross pathology were observed between the *C*. *jejuni* wild-type strain and the *ciaD* mutant. The *C*. *jejuni ciaD* complemented isolate partially restored the gross pathology to levels intermediate between the *C*. *jejuni* wild-type strain and *ciaD* mutant. **(B)** Ileocecocolic histopathologic lesion scores of all mice in the *in vivo* oral challenge study. Lesion scores for the *ciaD* mutant group and the *C*. *jejuni* wild-type group were significantly different, based on nonparametric Kruskal Wallis one-way ANOVA followed by the Mann–Whitney test for pairwise comparisons and the Holm-Šidák procedure for correction for multiple comparisons. Lesion scores in the *ciaD* complemented group showed that 3/13 (23%) of mice had restoration of the inflammatory phenotype. One mouse in the trypticase soy broth (TSB) treated group had spontaneous colitis. **(C)** Representative histopathology of ileocecocolic junctions. Bars represent 100 μm. Treatment groups were as follows: (1) mouse sham inoculated with trypticase soy broth, (2) mouse infected with the *C*. *jejuni* wild-type strain 11168, (3) mouse infected with the *ciaD* mutant, and (4) mouse infected with the *ciaD* complemented isolate. Note that the knockout of *ciaD* prevented tissue inflammation (panel 3) with tissues appearing similar to the TSB controls (panel 1); brackets in panels 1 and 3 show the normal crypt height. Mice receiving the *C*. *jejuni* wild-type strain (panel 2) had severe end stage typhlocolitis with significant increases in the crypt height of the lamina propria (upper bracket) and submucosa due to edema (lower bracket). Mononuclear cell infiltrates (dark purple cells) can be seen throughout the tissue. Complementation with *ciaD* restored the phenotype to that of severe typhlocolitis in the mouse shown in panel 4.

C57BL/6 IL-10^-/-^ mice were infected with a *C*. *jejuni* 11168 wild-type strain, a *ciaD* mutant, and a *ciaD* complemented isolate to assess the contribution of CiaD to the development of disease. Mice sham-inoculated with tryptic soya broth (TSB) were included as a negative control. We found that mice infected with the *C*. *jejuni ciaD* mutant exhibited less severe disease when compared to the *C*. *jejuni* wild-type strain, as judged by mouse survival (Additional file 9: Figure S9A), gross pathology (Figure 7A), histopathology (Figure 7B and C) and plasma IgG2b anti-*C*. *jejuni* antibody levels (Additional file 10: Figure S10). *C*. *jejuni* was only recovered at necropsy (either 35 or 36 days after inoculation) from mice inoculated with the wild-type strain (Additional file 9: Figure S9B); however, the fact that IL-10^-/-^ mice are unable to down-regulate inflammatory processes once they are initiated makes it possible to detect disease after pathogen clearance. Importantly, the *C*. *jejuni ciaD* mutant expressing a wild-type copy of *ciaD* produced more severe clinical signs of disease in some mice compared to the *ciaD* mutant (Figure 7A-C). The *C*. *jejuni ciaD* mutant produced no GI lesions, comparable to the TSB controls (Figure 7C, panels 1 and 3) and in contrast to the 11168 wild-type strain that produced severe end stage typhlocolitis with neutrophilic exudates, increased crypt height, crypt abscesses, mononuclear cell infiltration and massive subcutaneous edema (Figure 7C, panel 2). Specifically, the *ciaD* mutant expressing a wild-type copy of *ciaD* produced a marked increase in gross pathology (*i*.*e*., thickening of GI tract wall and/or enlarged ileocecocolic lymph node) and histopathology (*i.e.,* increased crypt height, goblet cell hyperplasia, and mononuclear cell infiltration) (Figure 7C, panel 4). Application of an established histopathologic scoring system to ileocecocolic sections from mice in all groups confirmed these observations (Figure 7B). Specifically, the *ciaD* mutant expressing a wild-type copy of CiaD exhibited a significant increase in gross pathology (*i*.*e*., thickening of GI tract wall and/or enlarged ileocecocolic lymph node). Theoretically, the *ciaD* complemented isolated would have achieved the same level of histopathology as the wild-type if it had been allowed to go longer. These results suggest that CiaD is contributing to the development of disease *in vivo*, but that colonization of IL-10^-/-^ mice is a multi-factorial process. Together, these data indicate that CiaD is necessary for the development of disease in the IL-10^-/-^ mouse model of campylobacteriosis.

## Discussion

In this study we identified and characterized a novel *C*. *jejuni* effector protein that is secreted and delivered to host cells via the flagellar T3SS. We found that CiaD is delivered to the cytosol of human INT 407 epithelial cells via a flagellar T3SS, where it is involved in maximal activation of the MAP kinase signaling pathways Erk 1/2 and p38. Interestingly, CiaD contains a MKD that is necessary for subversion of the host cell signaling pathways, leading to the secretion of IL-8 and invasion of host cells. MAP kinases are activated by the phosphorylation of their tyrosine and threonine residues by their specific MAP kinase kinase (MAPKK). The phosphorylation of MAP kinases are facilitated by a specific MKD motif, which allows the proper interaction to occur without crosstalk to inappropriate signaling molecules. We also report that CiaD is required for the development of acute disease and colon lesions.

MKD’s have been identified in several bacterial effector proteins, including SpvC, OspF, and VP1680 from *Salmonella enterica*, *Shigella spp*. and *V*. *parahaemolyticus*, respectively [33-36]. The SpvC and OspF MKD canonical (K/R)_1–2_-(X)_2–6_-O_A_-X-O_B_ sequence [37] are located near the N-terminus in a disordered region. Similarly, both proteins have been shown to interact with Erk 1/2 to facilitate the irreversible dephosphorylation of threonine residues crucial for Erk 1/2 activation [38]. However, the MKD is located centrally within a region of low-complexity in the *V*. *parahaemolyticus* effector VP1680. VP1680 is necessary for the activation of the p38 and Erk 1/2 signaling pathways [36]. Bioinformatics analysis of CiaD shows that the MKD is near the C-terminus in an area of low-complexity and is required for maximal activation of Erk 1/2 and p38. These data also suggest that there are two distinct classes of MKD containing effector proteins. One class inhibits the activation of these pathways (*i*.*e*., SpvC and OspF) and the other class stimulates the activation of the MAP kinases (*i*.*e*., VP1680 and CiaD). The CiaD (ΔS/T)P mutant induced the secretion of IL-8 and invaded host cells to a level that was indistinguishable from a *C*. *jejuni* wild-type strain. This finding suggests that CiaD does not need to be phosphorylated at this motif, but does not exclude the possibility CiaD is phosphorylated at another site.

Inhibition of either Erk 1/2 or p38 has been reported to lead to a significant decrease in the amount of *C*. *jejuni* internalized into host cells [23]. However, others have shown that these pathways do not affect bacterial invasion [18]. Our results support the findings that Erk 1/2 and p38 are necessary for maximal invasion by *C*. *jejuni*. While Erk 1/2 and p38 have also been shown to be necessary for invasion of *Shigella*, *Chlamydia*, and *Salmonella spp*. [39-41], the molecular mechanism by which Erk 1/2 and p38 promote bacterial invasion of intestinal cells is not known. It may be that the activation of Erk 1/2 and p38 alter gene transcription and the synthesis of proteins necessary for bacterial uptake or that Erk 1/2 and p38 activate other cellular proteins involved in bacterial invasion.

Acute inflammation caused by *C*. *jejuni* or other invasive enteric pathogens, such as *Shigella* spp. and *Salmonella enterica* serotype Typhimurium (*S*. Typhimurium), is associated with extensive epithelial injury and necrosis of the colon and the terminal ileum, with neutrophils being responsible for the tissue damage accompanying exudative inflammation. These processes all contribute to intestinal fluid accumulation and exudative diarrhea often with hemorrhage. Similarly, the inflammatory response is associated with changes in the gut microbiota, including an increased abundance of bacteria belonging to the family Enterobacteriaceae [42]. We show that the development of disease requires CiaD. The introduction of the wild-type copy of *ciaD* into the *ciaD* mutant resulted in greater gross- and histopathologic lesions than infection with the *ciaD* mutant, but somewhat less changes than the *C*. *jejuni* wild-type strain. One possible reason for this intermediate phenotype is that the expression of the wild-type copy of *ciaD* in the mutant may be altered. The partial restoration in virulence observed with the *C*. *jejuni ciaD* complemented isolate is consistent with the results of complementation experiments performed by others using *in vitro* challenge studies [14,43]. Noteworthy is that this is the first time that a *C*. *jejuni* effector protein has been found to be required for the development of disease in a mouse model. This is also in agreement with our previous results indicating that the Cia proteins contribute to the development of disease in piglets [14]. More specifically, infection of piglets with a *ciaB* mutant exhibited less severe disease as compared to a *C*. *jejuni* wild-type strain, as judged by clinical presentation and histopathology [14].

We discovered CiaD is required for the maximal invasion of human epithelial cells, induction of the inflammatory response, and the activation of the cellular signaling components Erk 1/2 and p38. We demonstrate that CiaD is necessary for the development of disease and colon inflammatory lesions *in vivo* using the IL-10 KO mouse model for campylobacteriosis. In addition, we observed a plasma IgG2b anti-*C*. *jejuni* antibody response in mice infected with the *C*. *jejuni* wild-type strain and complemented *ciaD* isolate, suggesting that *C*. *jejuni* stimulates a general proinflammatory response in IL-10^-/-^ mice. We believe that CiaD is, in part, responsible for the generation of plasma IgG2b anti-*C*. *jejuni* antibody response, as this response was not observed in mice inoculated with the *ciaD* mutant (Additional file 10: Figure S10). This finding is consistent with the fact that: 1) T-cell derived IL-10 is an inhibitor of the Th1 immune response, so an absence of IL-10 promotes a robust Th1 directed antibody response (predominantly IgG2b) [44]; and 2) The *C*. *jejuni* wild-type strain and complemented *ciaD* isolate are more invasive than the *ciaD* mutant. Noteworthy is that the Th1 “cell mediated” immune response is observed with invasive pathogens, including *C*. *jejuni*[44-47]. In conclusion, this is the first time that a *C*. *jejuni* effector protein has been shown to contribute to the development of acute disease. Future work will be directed towards the identification of a host cell-binding partner of CiaD, and to understand the role of Erk 1/2 and p38 in the larger scope of bacterial invasion.

## Methods

### Bacterial strains, tissues culture types, and plasmids

*Campylobacter jejuni* strains for *in vitro* experiments were cultured on Muller-Hinton agar plates supplemented with bovine citrated 5% (v/v) blood (MHB). Cultures for mouse inoculation studies were grown on trypticase soy agar containing 5% sheep’s blood. When appropriate, chloramphenicol (8 μg/ml) and tetracycline (2 μg/ml) were added to the media. Cultures were grown at 37°C in microaerobic conditions (5% O_2_, 10% CO_2_, and 85% N_2_). Cell lines used in this study were obtained from the American Type Tissue Culture Collection. INT 407 (ATCC CCL 6) and Caco-2 (ATCC HTB-37) cells were cultured in Minimal Essential Medium (MEM) supplemented with 10 mM sodium pyruvate, 20 mM glutamine, and 10% (v/v) fetal bovine serum (FBS). CT-26 (ATCC CRL-2638) cells were cultured in RPMI supplemented with 10% (v/v) fetal bovine serum (FBS). Plasmids and bacterial isolates used in this study are described in the Supplemental Methods (Additional file 1) and Additional file 11: Table S1. Primers used in this study are described in Supplemental Methods (Additional file 1) and Additional file 12: Table S2.

### Secretion assay

Secretion assays were performed as previously described [12]. Briefly, overnight cultures of *C*. *jejuni* grown in 0.05% DOC MH broth were harvested and a bacterial suspension was added to 3 mL MEM +1% FBS for a final OD_540_ of 0.3/ml. Cultures were then incubated at 37°C for 3 hr under microaerobic conditions. Following incubation, supernatants were concentrated 100-fold by precipitation with 4 volumes of ice-cold 1 mM HCl-acetone. Samples were separated by SDS-PAGE, transferred to polyvinylidene fluoride (PVDF) membranes, and blots were probed with antibodies against FLAG, CysM, and the ACD of *cyaA*.

### Cia protein delivery assays

Secretion assays were performed as previously described [12]. *C*. *jejuni* overnight cultures grown in 0.05% DOC MH broth were harvested, and the bacterial suspension was added to MEM + 1% FBS for a final OD_540_ of 0.015/ml. INT 407 cells were washed once with PBS and 1 mL of a 0.015 OD_540_ mixture was added to each well of the 24-well tissue culture tray. The trays were centrifuged for 5 min at 800 *× g* and incubated for 30 min at 37°C. The media in the wells were removed and the wells were washed 3 times with PBS followed by the addition of 95°C 0.1 M HCl. Each tray was boiled for 15 min by placing it on a platform directly above boiling water. Lysates were collected and transferred to a clean centrifuge tube. cAMP levels were assessed using the Direct Cyclic AMP Enzyme Immunoassay Kit (Assay Designs) according to manufacturer’s specifications.

### Immunoblot analysis, cellular inhibitors, antibodies, and densitometry analysis

Bacterial whole cell lysates, serum supernatant samples, and INT 407 cellular lysates were collected and analyzed by SDS-polyacrylamide gel electrophoresis. The proteins were transferred to a PVDF membrane and probed with the indicated antibodies. The antibodies used in this work are described in the Supplemental Methods (Additional file 1). Band intensity was quantified using a LAS 4000 mini (GE healthcare) and the Multi Gauge V3.0 (Fujifilm, Valhalla, NY) software package. Inhibitors were added to the cells 30 min prior to infection and maintained thorough the experiment. The Erk 1/2 (PD98059) and the p38 (SB202190) inhibitors were used at a concentration of 50 M. Cell death was quantified by trypan blue straining. No significant death was observed with any treatment conditions. *C*. *jejuni* was pretreated with chloramphenicol (1024 μg/ml) for 30 min prior to infection of host cells and maintained throughout the experiment. Chloramphenicol treatment did not effect bacterial viability. This concentration of chloramphenicol completely inhibited bacterial protein synthesis, as judged by ^35^[S]-methionine incorporation assays (not shown and [7]).

### Ectopic expression

EGFP plasmids were purified using the Qiagen Plasmid Purification Kit (Qiagen, Valencia, CA) according to the manufacturer’s protocols and normalized to 200 ng/μl. Purified plasmids where transfected into INT 407 cells seeded on glass coverslips at a confluencey of 3 × 10^5^. Transfections where performed using the Qiagen Effectene Transfection reagent (Qiagen, Valencia, CA), according to the manufacturer’s specifications.

### Invasion and motility assays

Binding and internalization assays [48] and motility assays [12] were performed as previously described.

### IL-8 quantification

Interleukin-8 levels in cellular supernatants were quantified with a commercial ELISA kit (OptEIA Set, Becton Dickinson, Cowley, Oxford, UK) using the manufacturer’s protocol. Briefly, cells were inoculated with 3 × 10^7^ bacteria and centrifuged for 5 min at 800 *× g* to promote cell contact. After 30 min of incubation, the cells were washed one time and fresh media was added to each well (containing inhibitors where indicated). The cells were incubated at 37°C for 24 hr and the media collected. Supernatants were frozen at -20°C or used immediately.

### Bioinformatics

Operon prediction was performed using MicrobesOnline [49]. Euckaryotic linear motif analysis was performed by query of the ELM website (http://elm.eu.org, [25]).

### Animals

All animal experiments were conducted according to NIH guidelines under Michigan State University Animal Use Form approval 06/012-107-00. Two replicate experiments were conducted. A breeding colony of C57BL/6 IL-10^-/-^ mice (B6.129P2-IL10^*tm1Cgn*^/J) was maintained in a specific-pathogen-free colony at MSU with monitoring for genotype and colitogenic bacteria as previously described [44]. For experiments, mice were transferred to the University Research Containment Facility at MSU where they were individually housed in filter top cages, on sterile food and water ad libitum.

### *C*. *jejuni* inoculation of IL-10^-/-^ mice

Mice 8–12 weeks old were infected with ~1 × 10^10^ cfu *C*. *jejuni* by oral gavage and observed daily for clinical signs using standardized scoring criteria as previously described [44]. Mice were humanely euthanized and necropsied promptly when clinical signs of severe disease developed or at thirty-five days post-infection. Blood samples were obtained by cardiac puncture immediately following death of the mouse. The gastrointestinal (GI) tract was removed in its entirety and placed on clean absorbent bench paper. Observations on gross pathological changes were recorded during necropsy.

### Gross pathology and histopathology

Gross pathology examination in all portions of the GI tract was performed by trained personnel as previously described [44]. The following criteria were used for scoring: no gross pathological changes (None, 0); either thickening of the GI tract wall or enlarged ileocecocolic lymph node (ICC or TW, 1); both thickening of GI tract wall and enlarged ileocecocolic lymph node (ICC and TW, 2); and bloody lumen contents in cecum or colon or both (BLC, 3). For histopathology, the ileocecocolic junction was removed and injected with 10% phosphate buffered formalin (Fisher Scientific, Pittsburgh, PA, USA), placed in a histological cassette and submerged in 10% phosphate buffered formalin. After 24 hr, the cassette was drained and transferred to 60% ethanol. Tissue processing, paraffin embedment, sectioning, and hematoxylin and eosin staining were carried out at the Investigative Histopathology Laboratory, Division of Human Pathology, Department of Physiology, Michigan State University. After randomization and coding to conceal identity, slides were read in a blinded fashion by a single investigator (LSM) according to a previously described scoring system [44].

### Plasma IgG2b anti-*C*. *jejuni* antibody

Plasma IgG2b anti-*C*. *jejuni* levels were determined via ELISA as previously described [44].

### Statistical analysis

All data was evaluated using a one-way ANOVA followed by post-hoc Tukey’s or Dunnet’s analysis of the means, using Prism 6 (GraphPad Software, La Jolla, CA). Statistical significance was defined by a maximum value of *P < 0.05 for all *in vitro* experiments and *P < 0.10 for all *in vivo* experiments. All experiments were performed a minimum of three times to ensure reproducibility. Kaplan Meier log rank analyses were performed using SigmaStat 3.1 (Systat Software, Port Richmond, CA) to assess the survival data. Gross pathology was analyzed using SigmaStat 3.1. The nonparametric Kruskal Wallis one-way ANOVA was used for gross pathology scoring. Scores for analysis of gross pathology data were assigned as follows: no gross pathological changes (None, 0); either thickening of GI tract wall or enlarged ileocecocolic lymph node (ICC or TW, 1); thickening of GI tract wall and enlarged ileocecocolic lymph node (ICC and TW, 2); and thickening of GI tract wall and enlarged ileocecocolic lymph node plus bloody lumen contents in cecum or colon or both (BLC, 3). Kruskal Wallis nonparametric one-way ANOVA was performed. Mann Whitney pairwise comparisons with Holm-Sidak correction for multiple comparisons was performed to evaluate the difference between the means of the samples, as described previously [44]. Plasma IgG2b anti-*C*. *jejuni* levels were evaluated with Kruskal Wallis one-way non-parametric ANOVA, followed by Mann Whitney pairwise comparisons with Holm-Sidak correction for multiple comparisons of the means.

### Supplemental information

The data sets supporting the results of this article are included within the article and its additional files.

## Abbreviations

Cia: *Campylobacter* invasion antigens; Erk 1/2: Extracellular regulated kinase.

## Competing interests

The authors declare that they have no competing interests

## Authors’ contributions

DRS planned, performed, and wrote the manuscript. MEK and LSM planned experiments, analyzed data and contributed to manuscript preparation. TPE, LD, JAB planned and performed experiments. All authors read and approved the final manuscript.

## Supplementary Material

Additional file 1: Figure S1 The *C*. *jejuni ciaD* mutant secretes a known effector protein. (A) The *ciaD* mutant is Cia secretion competent. A *C*. *jejuni ciaD* mutant was transformed with the pRY111 vector harboring CiaD, CiaC and MetK fused to the ACD and the isolates were analyzed by immunoblot analysis. Supernatant and whole cell lysates were separated by SDS-PAGE, proteins transferred to PVDF membranes, and blots probed with an ACD antibody and CysM antibody. A *C*. *jejuni* wild-type strain without a plasmid and the MetK-ACD (S-adenosylmethionine synthetase) protein, which is localized in the bacterial cytoplasm, were included as negative controls. Molecular mass standards, in kilodaltons (kDa), are indicated on the left. Arrows indicate the CiaD-ACD and CiaC-ACD secreted proteins. (B) The *C*. *jejuni* CiaC-ACD, CiaD-ACD, and CiaC-ACD fusion proteins are synthesized in similar levels. The *C*. *jejuni* wild-type strain, *ciaD*, and a *flgBC* mutant transformed with the pRY111 vector harboring CiaD, CiaC and MetK fused to the ACD were analyzed by immunoblot analysis. Protein levels were quantified by BCA, normalized to ensure equal loading, separated by SDS-PAGE, transferred to PVDF membranes, and blots probed with an ACD antibody. Click here for file

Additional file 2: Figure S2 *C*. *jejuni* requires *de novo* protein synthesis for bacterial invasion and induction of secretion. (A) Pre-treatment of *C*. *jejuni* with chloramphenicol inhibits INT 407 cell invasion. *C*. *jejuni* were pretreated with chloramphenicol (1024 μg/mL) for 30 min prior to infection of INT 407 cells. Cell invasion was assessed using a gentamicin-protection assay as outlined in Supplemental Methods (Additional file 1). (B) *C*. *jejuni* requires *de novo* protein synthesis for maximal IL-8 secretion. An IL-8 secretion time course assay was performed by infecting INT 407 cells with a *C*. *jejuni* wild-type strain that had been pretreated for 30 min with chloramphenicol (1024 μg/mL) and harvesting the supernatants at various times post-infection. IL-8 in the supernatant samples was quantified by ELISA as described in Methods. Gray bars indicate IL-8 quantities from INT 407 cells infected with an untreated *C*. *jejuni* wild-type strain. The black bars indicate IL-8 quantities from INT 407 cells infected with a *C*. *jejuni* wild-type strain that was pre-treated with chloramphenicol. The asterisks indicate the time points (4 and 6 hr) at which there are significant differences in the amount of IL-8 produced compared to the untreated samples, as judged by one-way ANOVA followed by post-hoc Tukey’s analysis (*P* < 0.05). Error bars represent ± SEM. Click here for file

Additional file 3: Figure S3 Ectopic expression of CiaD in host INT 407 cells induces IL-8 secretions. INT 407 cells were transfected with CiaD-EGFP and EGFP-only eukaryotic expression vectors. Cells treated with the transfection reagent Effectene were also included as a vehicle control. IL-8 levels were assessed by ELISA 24 hr following transfection. The asterisks indicate that the amount of IL-8 produced was significantly increased compared to the EGFP-only control, as judged by student’s *t*-test (*P* < 0.05). Error bars represent ± SEM. Click here for file

Additional file 4: Figure S4 Mutation of Cj0789, which is the gene downstream of *ciaD*, does not have an effect on IL-8 secretion, indicating the Cj0788 mutation is not polar. INT 407 cells were infected with *C*. *jejuni* for 24 hr. Following infection, supernatants were collected and IL-8 levels quantified using an IL-8 ELISA. The asterisks indicate that the amount of IL-8 produced was significantly decreased compared to the *C*. *jejuni* wild-type strains (F38011 and NCTC 11168), as judged by one-way ANOVA followed by post-hoc Tukey’s analysis (*P* < 0.05). Error bars represent ± SEM. Click here for file

Additional file 5: Figure S5 MAP kinase inhibition leads to an additive effect in IL-8 secretion by the *C*. *jejuni ciaD* mutant. Inhibitors to Erk 1/2 and p38 were added to INT 407 cells for 30 min prior to the addition of the *C*. *jejuni ciaD* mutant. The *C*. *jejuni* wild-type strain was included as a positive control. The mean value calculated for ‘cells only’ was subtracted from all other values. The asterisk indicates a significant reduction in the amount of IL-8 secreted form INT 407 cells infected with the *ciaD* mutant in the presence of the Erk 1/2 and p38 inhibitors as compared to the value obtained for the untreated INT 407 cells infected with the *C*. *jejuni ciaD* mutant, as judged by one-way ANOVA followed by post-hoc Tukey’s analysis (*P* < 0.05). Error bars represent ± SEM. Click here for file

Additional file 6: Figure S6 The pro-inflammatory cytokine IL-8 is not required for bacterial invasion. (A) Caco-2 cells were infected with *C*. *jejuni* for 30 min followed by the addition of 300 pg/ml of IL-8 to the *C*. *jejuni ciaD* mutant and wild-type strain. The bars represent the mean of bacterial invasion of the wild-type and the *ciaD* mutant with the addition of IL-8 or no treatment. (B) The activation status of Akt was determined via immunoblot to confirm IL-8 induced signaling. 300 pg/ml of IL-8 was added to INT 407 cells for 15 min and cellular lysates were prepared. Blots were probed with phospho-specific antibodies to Akt (*M*_r_ = 62 kDa). All blots were stripped and re-probed with an anti-Akt (*M*_r_ = 60 kDa) antibody. The asterisk indicates a significant decrease compared to the *C*. *jejuni* wild-type strain, as judged by one-way ANOVA followed by post-hoc Tukey’s analysis (*P* < 0.05). Error bars represent ± SEM. Click here for file

Additional file 7: Figure S7 The *C*. *jejuni ciaD* mutant has reduced MAP kinase signaling. (A) Maximal activation of MAP kinase signaling requires CiaD. The activation status of the MAP kinase signaling components was determined using a phospho-spot array assay as outlined in Supplemental Methods (Additional file 1). INT 407 cells were infected with the *C*. *jejuni* wild-type strain and *C*. *jejuni ciaD* mutant for 3 hr. Cellular lysates were assayed using the spot array. Pictured are the spot array profiles of the *C*. *jejuni* wild-type and *C*. *jejuni ciaD* mutant. (B) Maximal activation of MAP kinase signaling requires CiaD. Densitometry was performed on the phospho-spot arrays performed in Panel B. Significance was not assessed, as this experiment was used as a screen for activation. Click here for file

Additional file 8: Figure S8 The CiaD ΔMKD site and Δ(S/T)P proteins are synthesized and the isolates that produce these variant proteins are motile. (A) Deletion of the MKD site and the (S/T)P site does not significantly effect protein synthesis. The *C*. *jejuni ciaD* mutant transformed with a pRY111 vector encoding either a wild-type copy of the CiaD protein, the MAP kinase docking motif (ΔMKD site) mutant protein, or the (Δ(S/T)P) mutant protein fused to a FLAG-tag were analyzed by immunoblot analysis. The blots were probed with a FLAG antibody. Blots were also stripped and re-probed with an anti-CysM antibody to ensure equal loading of each sample. (B) *C*. *jejuni* strains synthesizing the CiaD MAP kinase docking motif (ΔMKD site) mutant protein and CiaD proline directed-phosphorylation site (Δ(S/T)P) mutant protein are motile. Click here for file

Additional file 9: Figure S9 *C*. *jejuni* colonization of IL10^-/-^ mice. (A) Percent survival of C57BL/6 IL-10^-/-^ mice infected with the *C*. *jejuni* 11168 wild-type strain, *ciaD* mutant, and the *ciaD* complemented isolate. No significant differences were observed in the number of surviving mice as judged by Kaplan Meier log rank analysis. The mouse inoculated with the *C*. *jejuni ciaD* complemented isolate that died 8 days post-infection was excluded from the gross pathology evaluation (Figure 7A), as the cause of death was not known. (B) Colonization of mice infected with the *C*. *jejuni* wild-type strain, *ciaD* mutant, and the *ciaD* complement isolate was assessed 35 days post-infection by bacterial CFU determination of colon content and *Campylobacter* PCR on DNA extracted from frozen cecal tips of all mice that were negative by culture. Click here for file

Additional file 10: Figure S10 *C*. *jejuni* stimulates the production of plasma IgG2b. Plasma IgG2b anti-*C*. *jejuni* antibody levels in mice infected with a *C*. *jejuni* wild-type strain, *ciaD* mutant, and the *ciaD* complemented isolate. Levels of IgG2B were evaluated via ELISA. We found that the *C*. *jejuni* wild-type strain had a significant increase in the amount of detectable IgG2b as judged by nonparametric Kruskal Wallis one-way ANOVA, followed by post hoc comparisons using Mann Whitney pairwise comparisons. Corrections were made for multiple comparisons using the Holm-Šidák test. Click here for file

Additional file 11: Table S1 Bacterial isolates and plasmids used in this study. Click here for file

Additional file 12: Table S2 Primers used in this study. Click here for file
