# Supplementary figures and images for: The Campylobacter jejuni CiaD effector protein activates MAP kinase signaling pathways and is required for the development of disease
Source: Cell Commun Signal. 2013 Oct 21;11:79. doi: 10.1186/1478-811X-11-79 (PMC3833307; doi:10.1186/1478-811X-11-79)

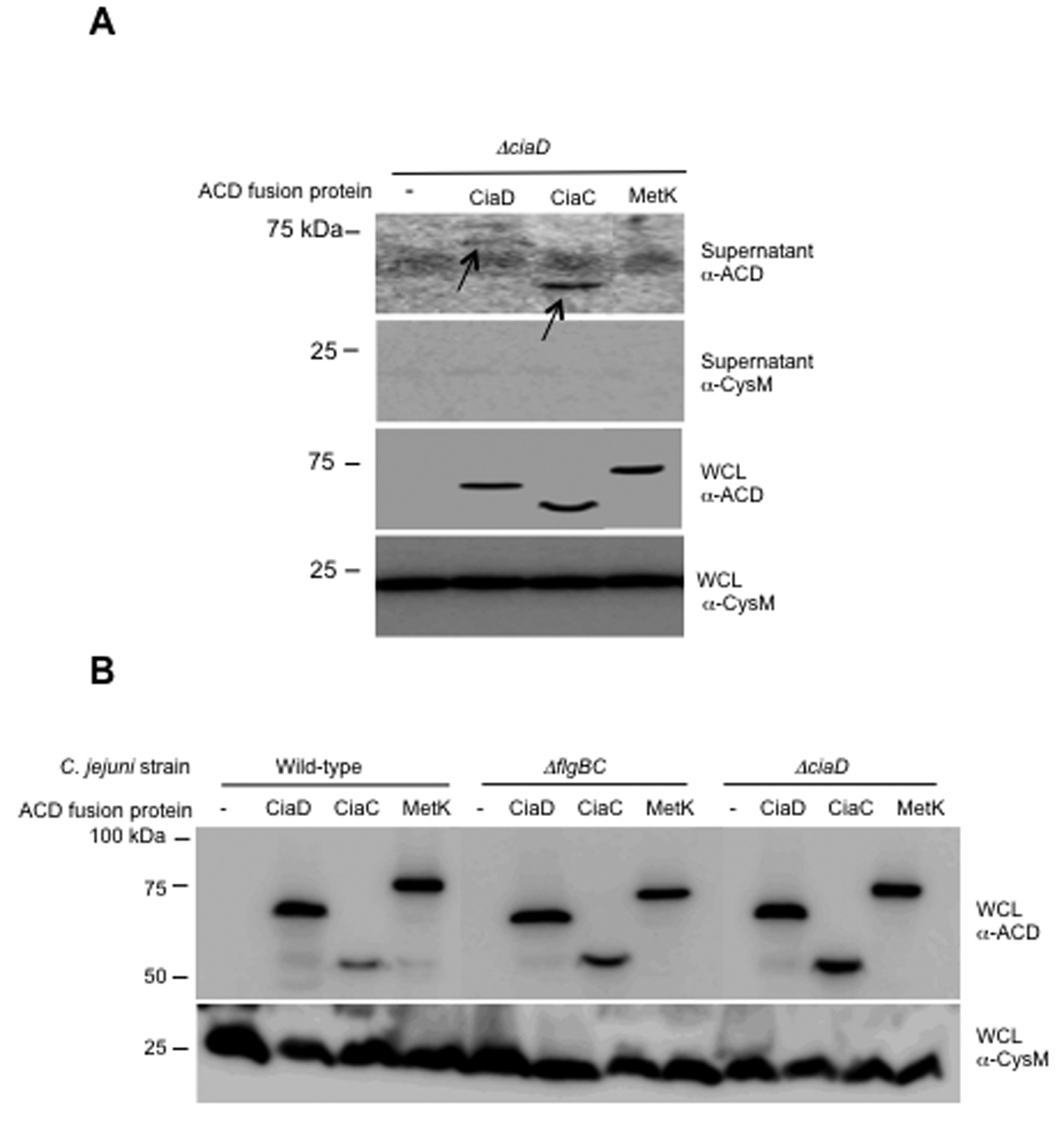

Supplement: Additional file 1: Figure S1 — The C. jejuni ciaD mutant secretes a known effector protein. (A) The ciaD mutant is Cia secretion competent. A C. jejuni ciaD mutant was transformed with the pRY111 vector harboring CiaD, CiaC and MetK fused to the ACD and the isolates were analyzed by immunoblot analysis. Supernatant and whole cell lysates were separated by SDS-PAGE, proteins transferred to PVDF membranes, and blots probed with an ACD antibody and CysM antibody. A C. jejuni wild-type strain without a plasmid and the MetK-ACD (S-adenosylmethionine synthetase) protein, which is localized in the bacterial cytoplasm, were included as negative controls. Molecular mass standards, in kilodaltons (kDa), are indicated on the left. Arrows indicate the CiaD-ACD and CiaC-ACD secreted proteins. (B) The C. jejuni CiaC-ACD, CiaD-ACD, and CiaC-ACD fusion proteins are synthesized in similar levels. The C. jejuni wild-type strain, ciaD, and a flgBC mutant transformed with the pRY111 vector harboring CiaD, CiaC and MetK fused to the ACD were analyzed by immunoblot analysis. Protein levels were quantified by BCA, normalized to ensure equal loading, separated by SDS-PAGE, transferred to PVDF membranes, and blots probed with an ACD antibody. [file 1478-811X-11-79-S1.tiff]

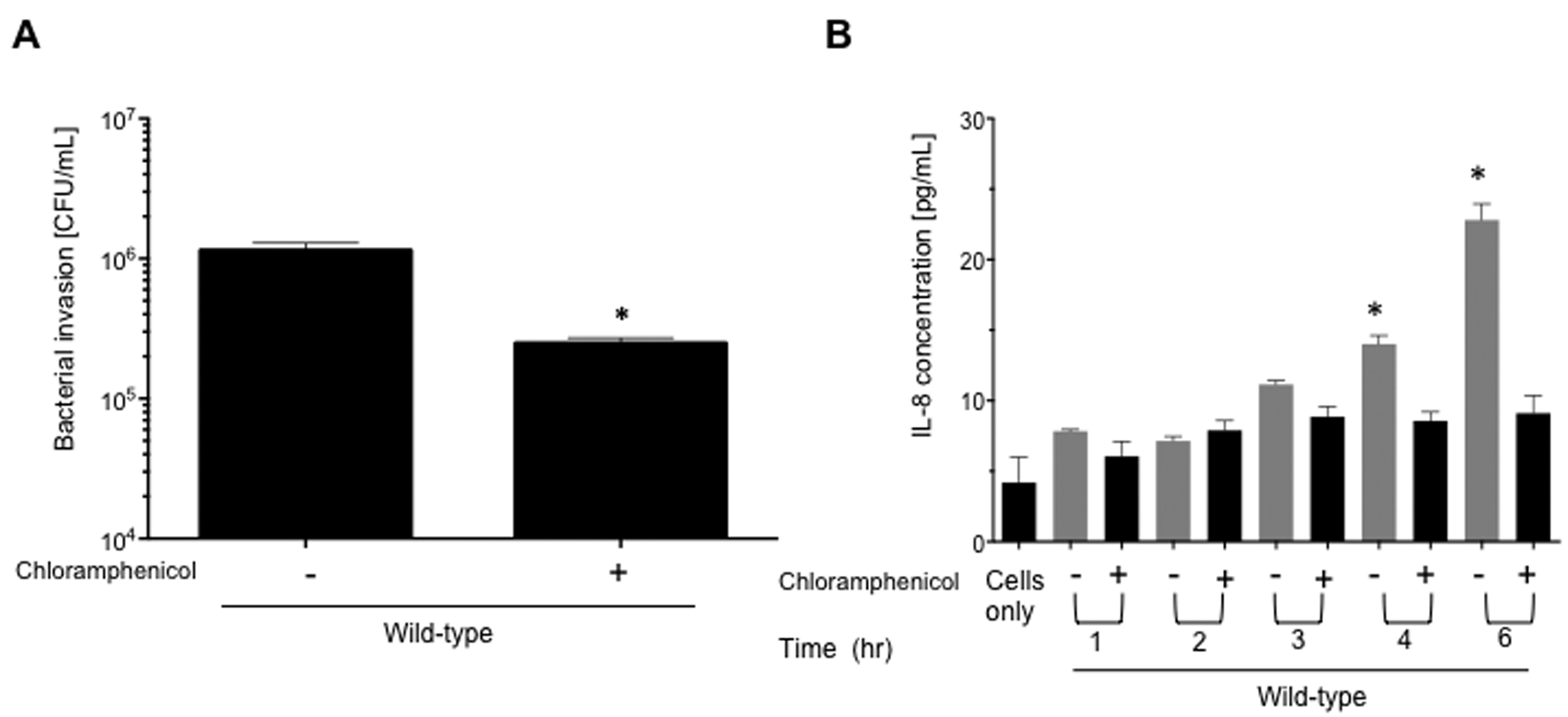

Supplement: Additional file 2: Figure S2 — C. jejuni requires de novo protein synthesis for bacterial invasion and induction of secretion. (A) Pre-treatment of C. jejuni with chloramphenicol inhibits INT 407 cell invasion. C. jejuni were pretreated with chloramphenicol (1024 μg/mL) for 30 min prior to infection of INT 407 cells. Cell invasion was assessed using a gentamicin-protection assay as outlined in Supplemental Methods (Additional file 1). (B) C. jejuni requires de novo protein synthesis for maximal IL-8 secretion. An IL-8 secretion time course assay was performed by infecting INT 407 cells with a C. jejuni wild-type strain that had been pretreated for 30 min with chloramphenicol (1024 μg/mL) and harvesting the supernatants at various times post-infection. IL-8 in the supernatant samples was quantified by ELISA as described in Methods. Gray bars indicate IL-8 quantities from INT 407 cells infected with an untreated C. jejuni wild-type strain. The black bars indicate IL-8 quantities from INT 407 cells infected with a C. jejuni wild-type strain that was pre-treated with chloramphenicol. The asterisks indicate the time points (4 and 6 hr) at which there are significant differences in the amount of IL-8 produced compared to the untreated samples, as judged by one-way ANOVA followed by post-hoc Tukey’s analysis (P < 0.05). Error bars represent ± SEM. [file 1478-811X-11-79-S2.tiff]

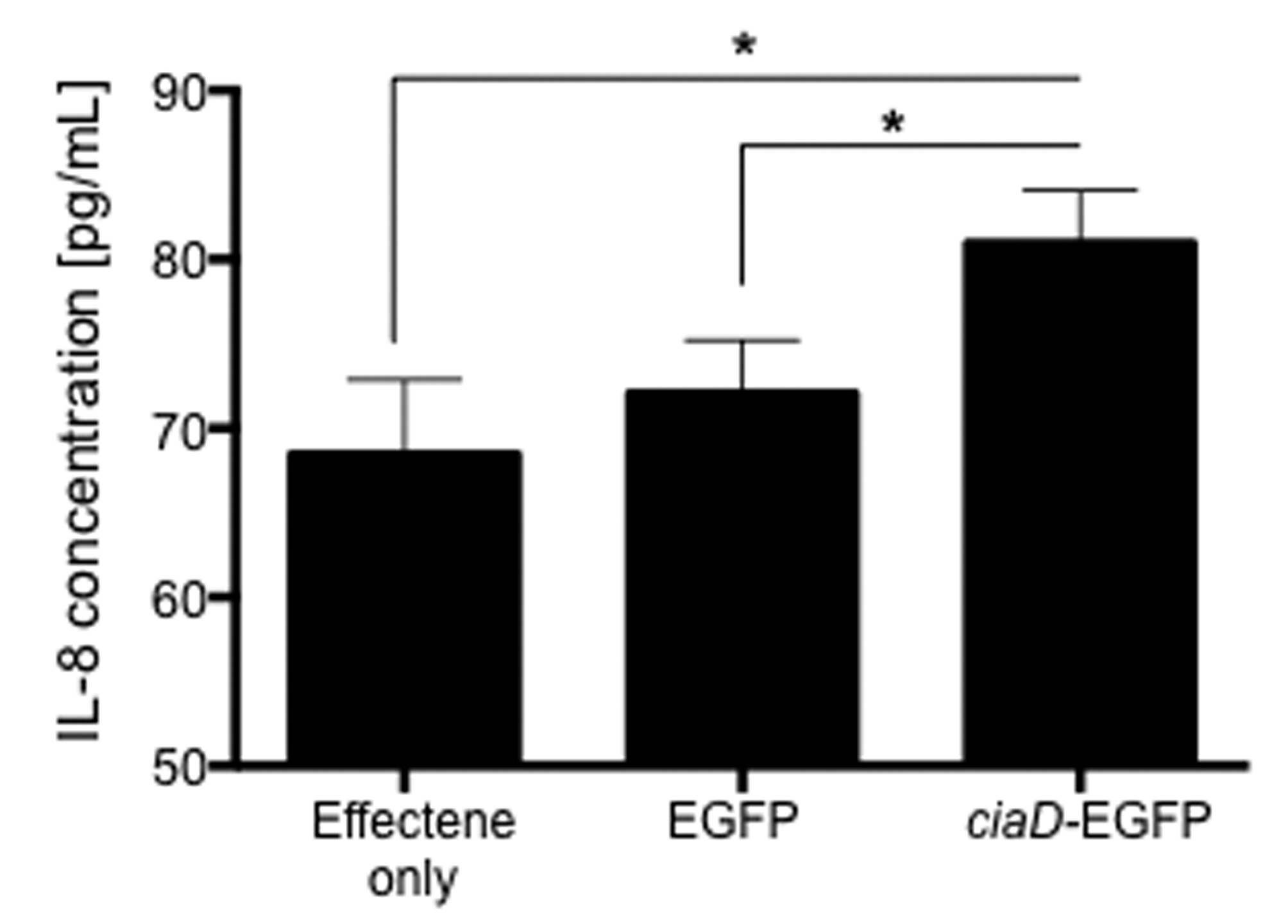

Supplement: Additional file 3: Figure S3 — Ectopic expression of CiaD in host INT 407 cells induces IL-8 secretions. INT 407 cells were transfected with CiaD-EGFP and EGFP-only eukaryotic expression vectors. Cells treated with the transfection reagent Effectene were also included as a vehicle control. IL-8 levels were assessed by ELISA 24 hr following transfection. The asterisks indicate that the amount of IL-8 produced was significantly increased compared to the EGFP-only control, as judged by student’s t-test (P < 0.05). Error bars represent ± SEM. [file 1478-811X-11-79-S3.tiff]

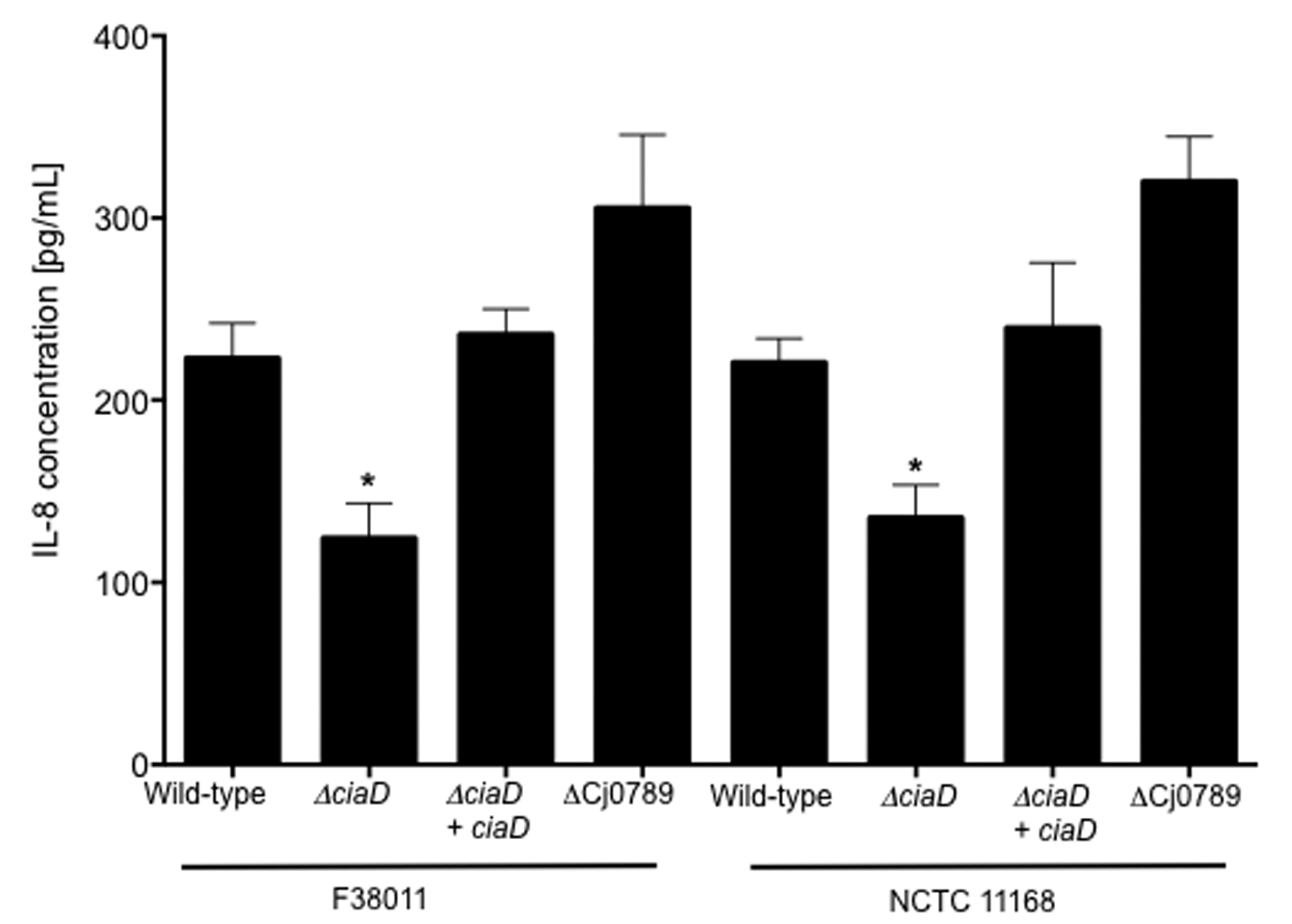

Supplement: Additional file 4: Figure S4 — Mutation of Cj0789, which is the gene downstream of ciaD, does not have an effect on IL-8 secretion, indicating the Cj0788 mutation is not polar. INT 407 cells were infected with C. jejuni for 24 hr. Following infection, supernatants were collected and IL-8 levels quantified using an IL-8 ELISA. The asterisks indicate that the amount of IL-8 produced was significantly decreased compared to the C. jejuni wild-type strains (F38011 and NCTC 11168), as judged by one-way ANOVA followed by post-hoc Tukey’s analysis (P < 0.05). Error bars represent ± SEM. [file 1478-811X-11-79-S4.tiff]

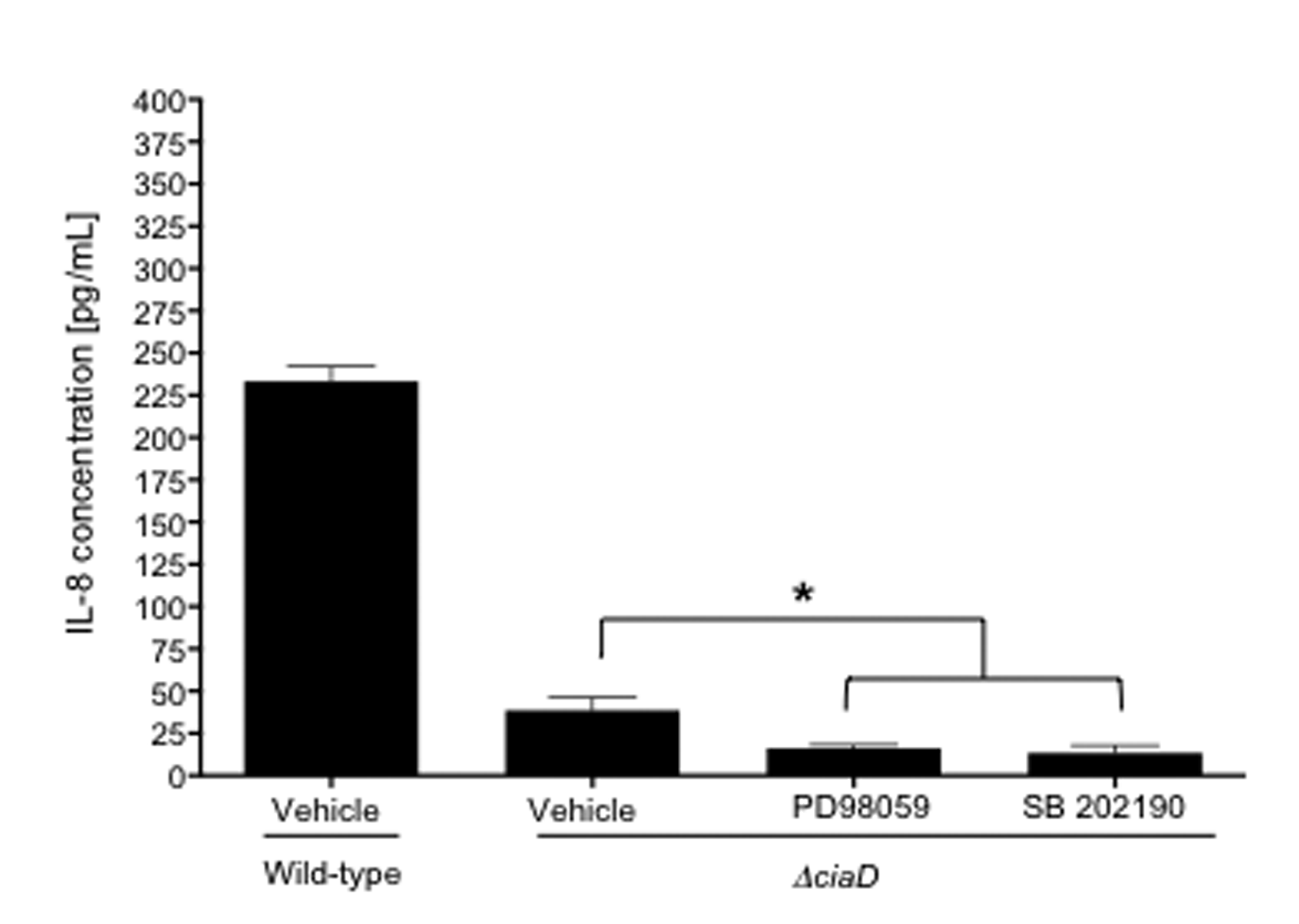

Supplement: Additional file 5: Figure S5 — MAP kinase inhibition leads to an additive effect in IL-8 secretion by the C. jejuni ciaD mutant. Inhibitors to Erk 1/2 and p38 were added to INT 407 cells for 30 min prior to the addition of the C. jejuni ciaD mutant. The C. jejuni wild-type strain was included as a positive control. The mean value calculated for ‘cells only’ was subtracted from all other values. The asterisk indicates a significant reduction in the amount of IL-8 secreted form INT 407 cells infected with the ciaD mutant in the presence of the Erk 1/2 and p38 inhibitors as compared to the value obtained for the untreated INT 407 cells infected with the C. jejuni ciaD mutant, as judged by one-way ANOVA followed by post-hoc Tukey’s analysis (P < 0.05). Error bars represent ± SEM. [file 1478-811X-11-79-S5.tif]

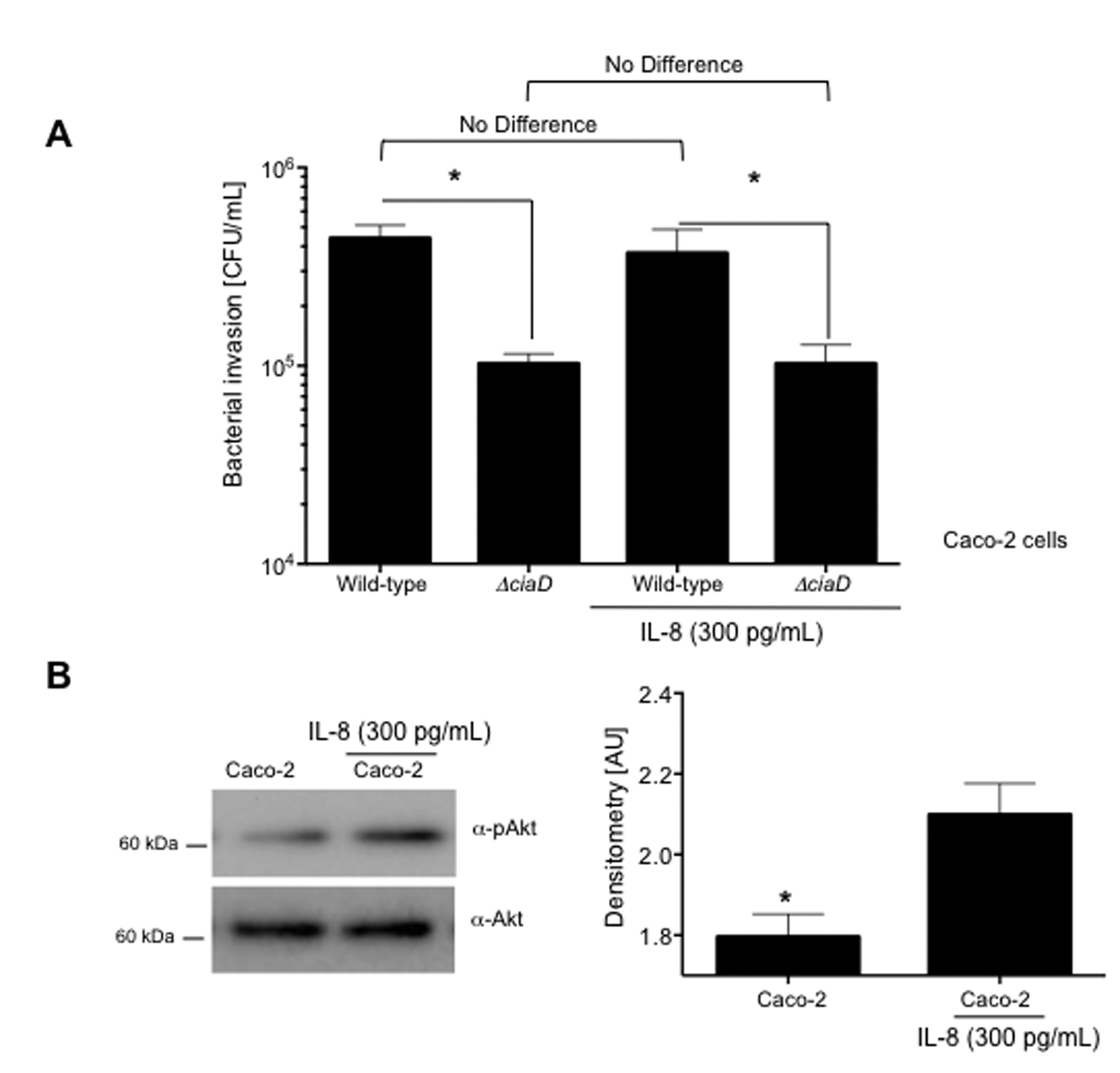

Supplement: Additional file 6: Figure S6 — The pro-inflammatory cytokine IL-8 is not required for bacterial invasion. (A) Caco-2 cells were infected with C. jejuni for 30 min followed by the addition of 300 pg/ml of IL-8 to the C. jejuni ciaD mutant and wild-type strain. The bars represent the mean of bacterial invasion of the wild-type and the ciaD mutant with the addition of IL-8 or no treatment. (B) The activation status of Akt was determined via immunoblot to confirm IL-8 induced signaling. 300 pg/ml of IL-8 was added to INT 407 cells for 15 min and cellular lysates were prepared. Blots were probed with phospho-specific antibodies to Akt (Mr = 62 kDa). All blots were stripped and re-probed with an anti-Akt (Mr = 60 kDa) antibody. The asterisk indicates a significant decrease compared to the C. jejuni wild-type strain, as judged by one-way ANOVA followed by post-hoc Tukey’s analysis (P < 0.05). Error bars represent ± SEM. [file 1478-811X-11-79-S6.tiff]

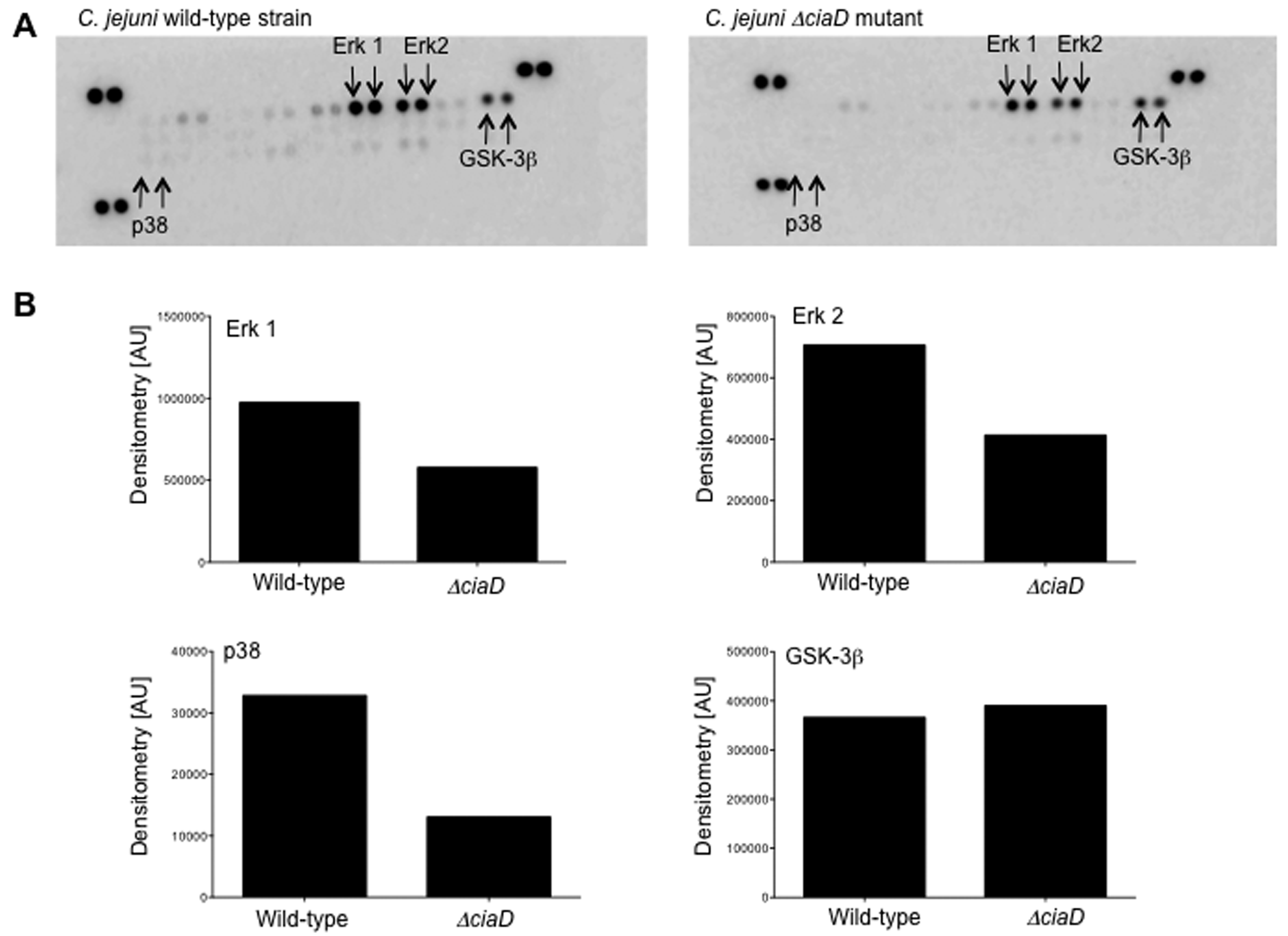

Supplement: Additional file 7: Figure S7 — The C. jejuni ciaD mutant has reduced MAP kinase signaling. (A) Maximal activation of MAP kinase signaling requires CiaD. The activation status of the MAP kinase signaling components was determined using a phospho-spot array assay as outlined in Supplemental Methods (Additional file 1). INT 407 cells were infected with the C. jejuni wild-type strain and C. jejuni ciaD mutant for 3 hr. Cellular lysates were assayed using the spot array. Pictured are the spot array profiles of the C. jejuni wild-type and C. jejuni ciaD mutant. (B) Maximal activation of MAP kinase signaling requires CiaD. Densitometry was performed on the phospho-spot arrays performed in Panel B. Significance was not assessed, as this experiment was used as a screen for activation. [file 1478-811X-11-79-S7.tiff]

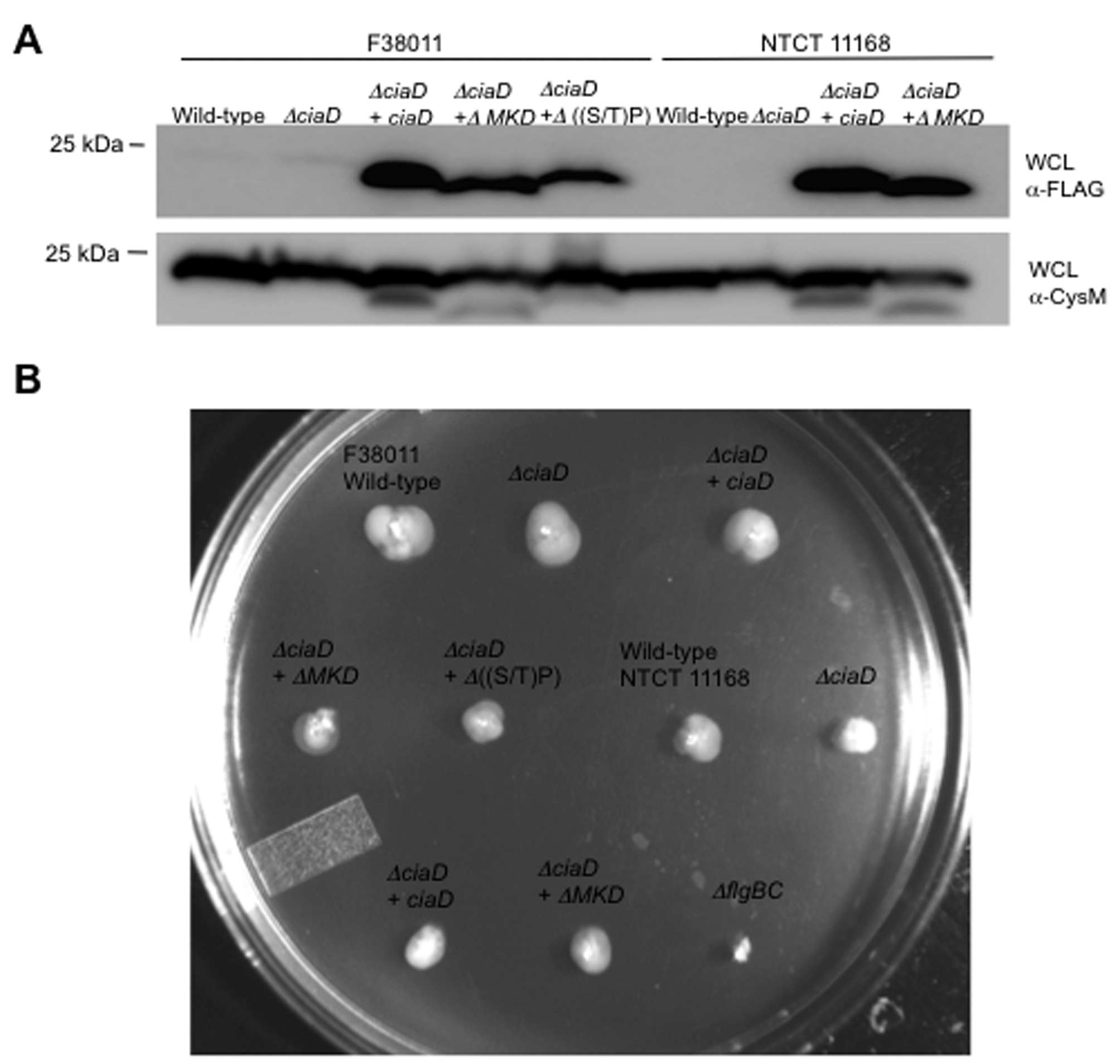

Supplement: Additional file 8: Figure S8 — The CiaD ΔMKD site and Δ(S/T)P proteins are synthesized and the isolates that produce these variant proteins are motile. (A) Deletion of the MKD site and the (S/T)P site does not significantly effect protein synthesis. The C. jejuni ciaD mutant transformed with a pRY111 vector encoding either a wild-type copy of the CiaD protein, the MAP kinase docking motif (ΔMKD site) mutant protein, or the (Δ(S/T)P) mutant protein fused to a FLAG-tag were analyzed by immunoblot analysis. The blots were probed with a FLAG antibody. Blots were also stripped and re-probed with an anti-CysM antibody to ensure equal loading of each sample. (B) C. jejuni strains synthesizing the CiaD MAP kinase docking motif (ΔMKD site) mutant protein and CiaD proline directed-phosphorylation site (Δ(S/T)P) mutant protein are motile. [file 1478-811X-11-79-S8.tiff]

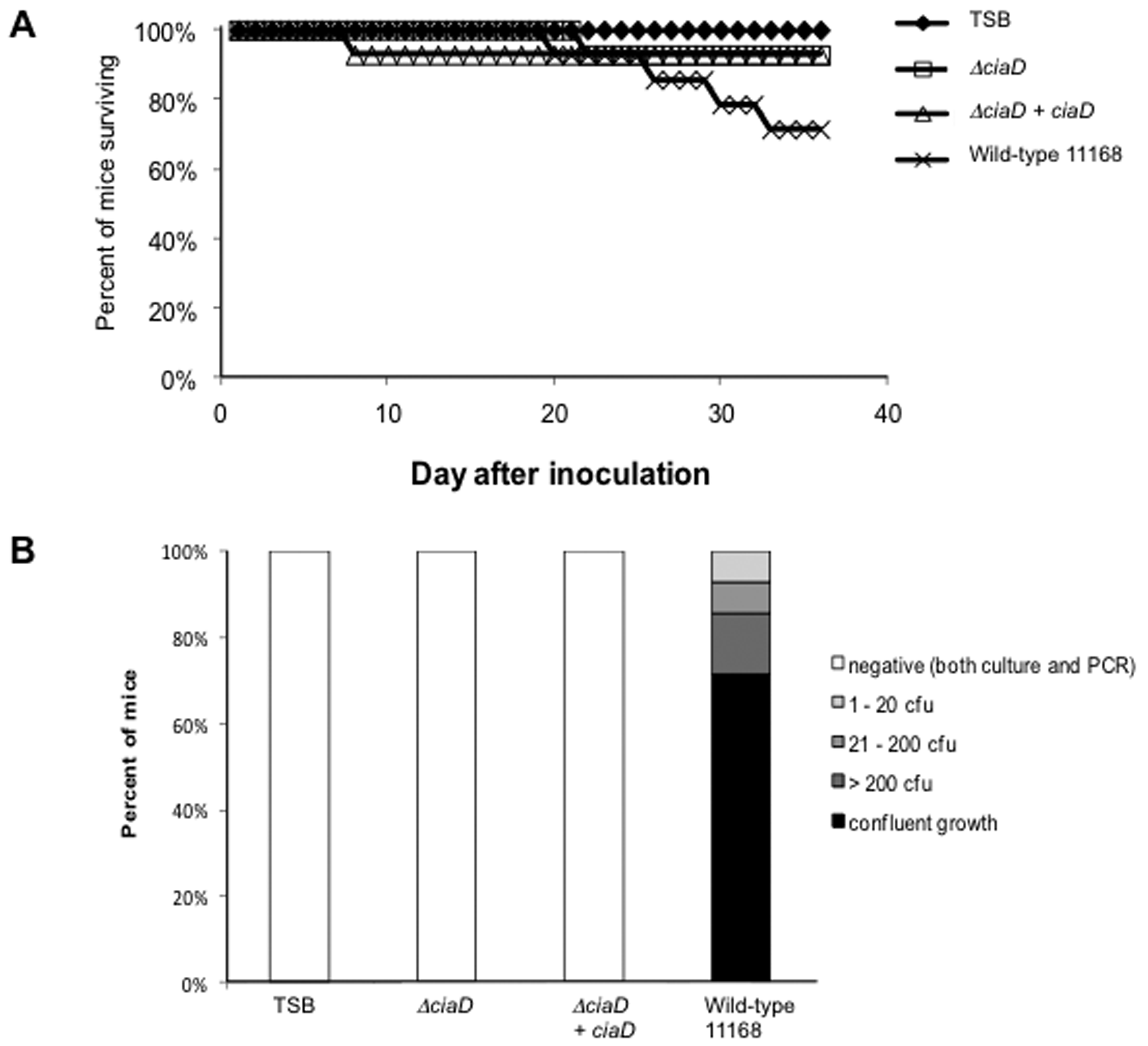

Supplement: Additional file 9: Figure S9 — C. jejuni colonization of IL10-/- mice. (A) Percent survival of C57BL/6 IL-10-/- mice infected with the C. jejuni 11168 wild-type strain, ciaD mutant, and the ciaD complemented isolate. No significant differences were observed in the number of surviving mice as judged by Kaplan Meier log rank analysis. The mouse inoculated with the C. jejuni ciaD complemented isolate that died 8 days post-infection was excluded from the gross pathology evaluation (Figure 7A), as the cause of death was not known. (B) Colonization of mice infected with the C. jejuni wild-type strain, ciaD mutant, and the ciaD complement isolate was assessed 35 days post-infection by bacterial CFU determination of colon content and Campylobacter PCR on DNA extracted from frozen cecal tips of all mice that were negative by culture. [file 1478-811X-11-79-S9.tiff]

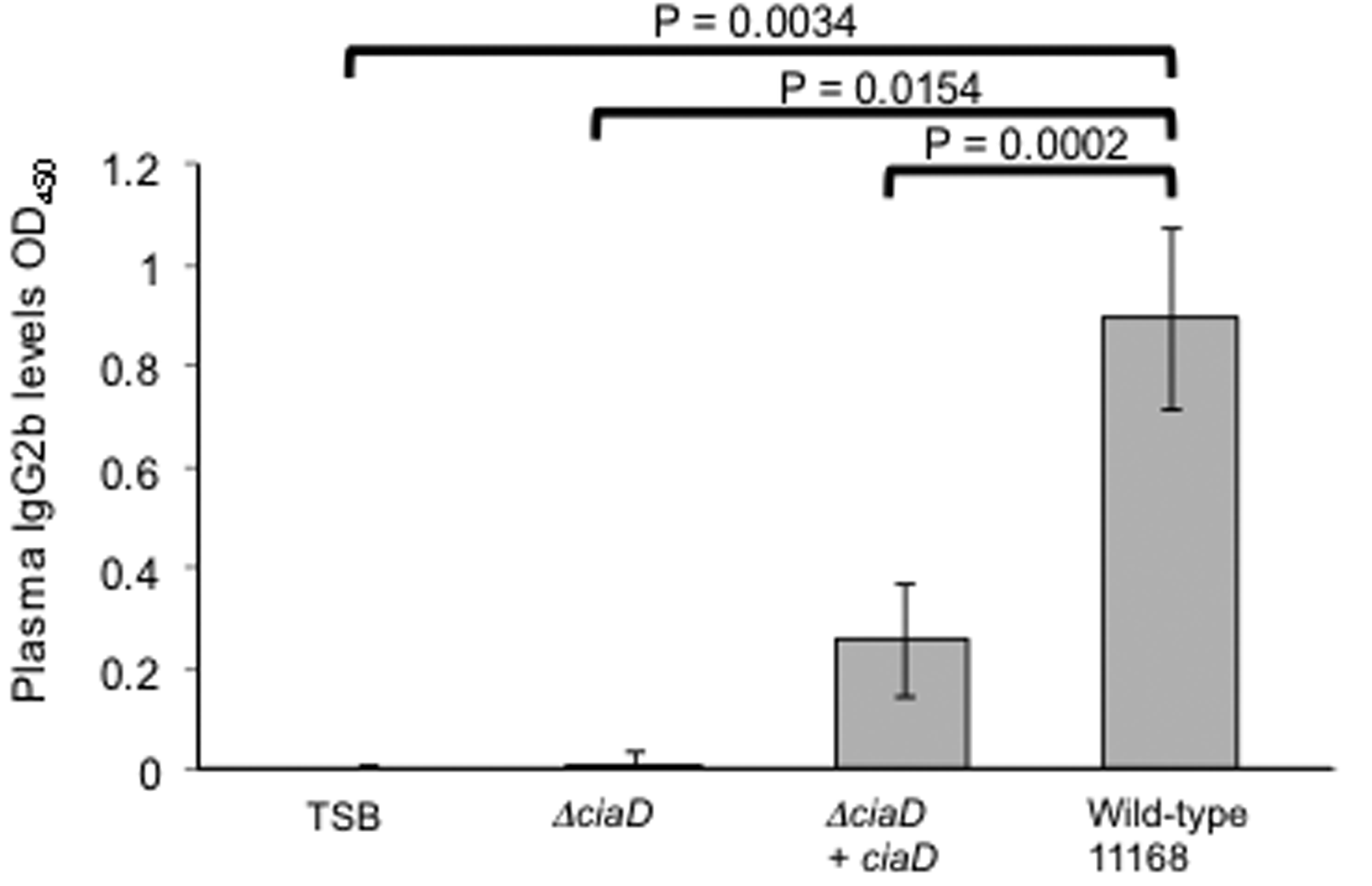

Supplement: Additional file 10: Figure S10 — C. jejuni stimulates the production of plasma IgG2b. Plasma IgG2b anti-C. jejuni antibody levels in mice infected with a C. jejuni wild-type strain, ciaD mutant, and the ciaD complemented isolate. Levels of IgG2B were evaluated via ELISA. We found that the C. jejuni wild-type strain had a significant increase in the amount of detectable IgG2b as judged by nonparametric Kruskal Wallis one-way ANOVA, followed by post hoc comparisons using Mann Whitney pairwise comparisons. Corrections were made for multiple comparisons using the Holm-Šidák test. [file 1478-811X-11-79-S10.tiff]

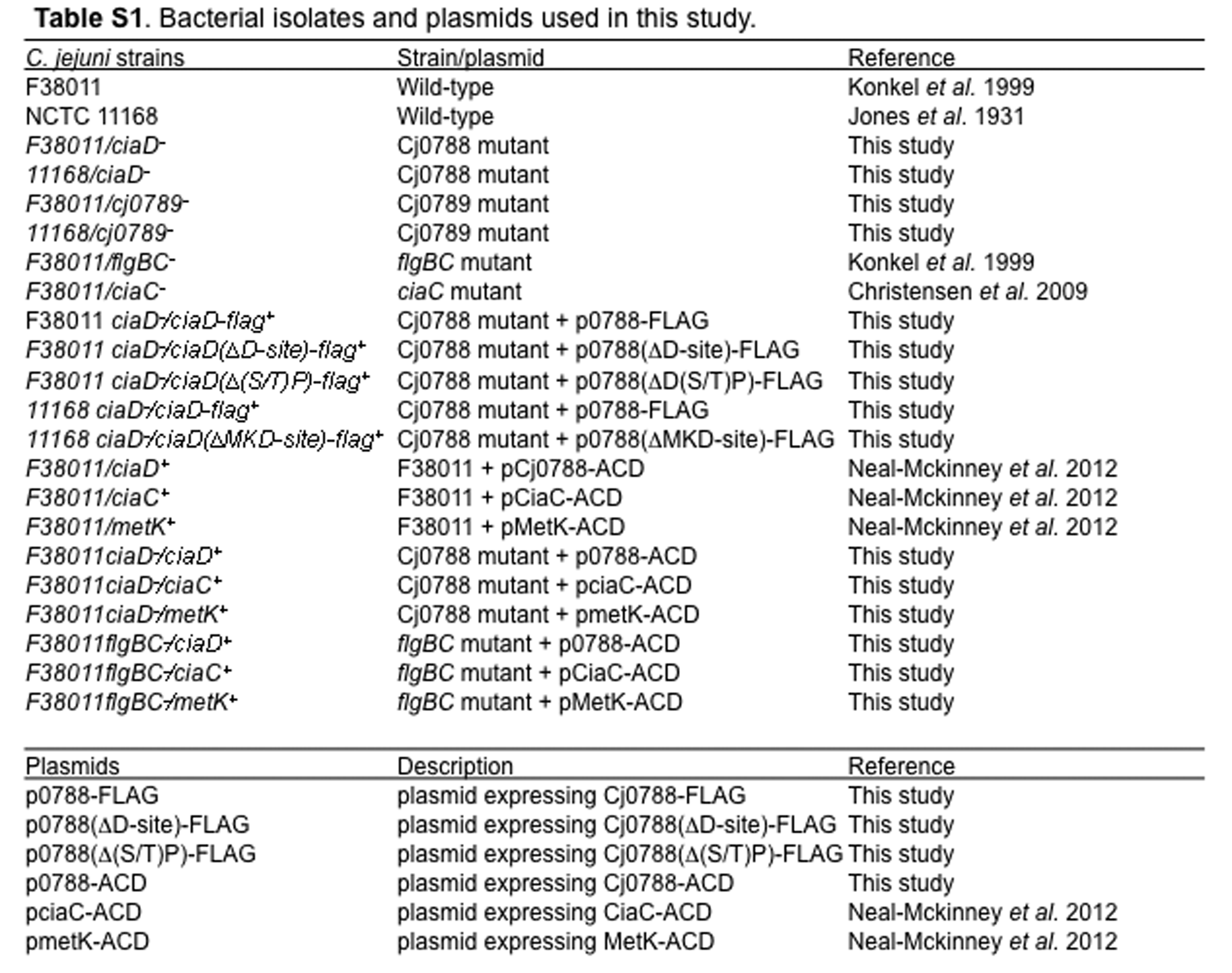

Supplement: Additional file 11: Table S1 — Bacterial isolates and plasmids used in this study. [file 1478-811X-11-79-S11.tiff]

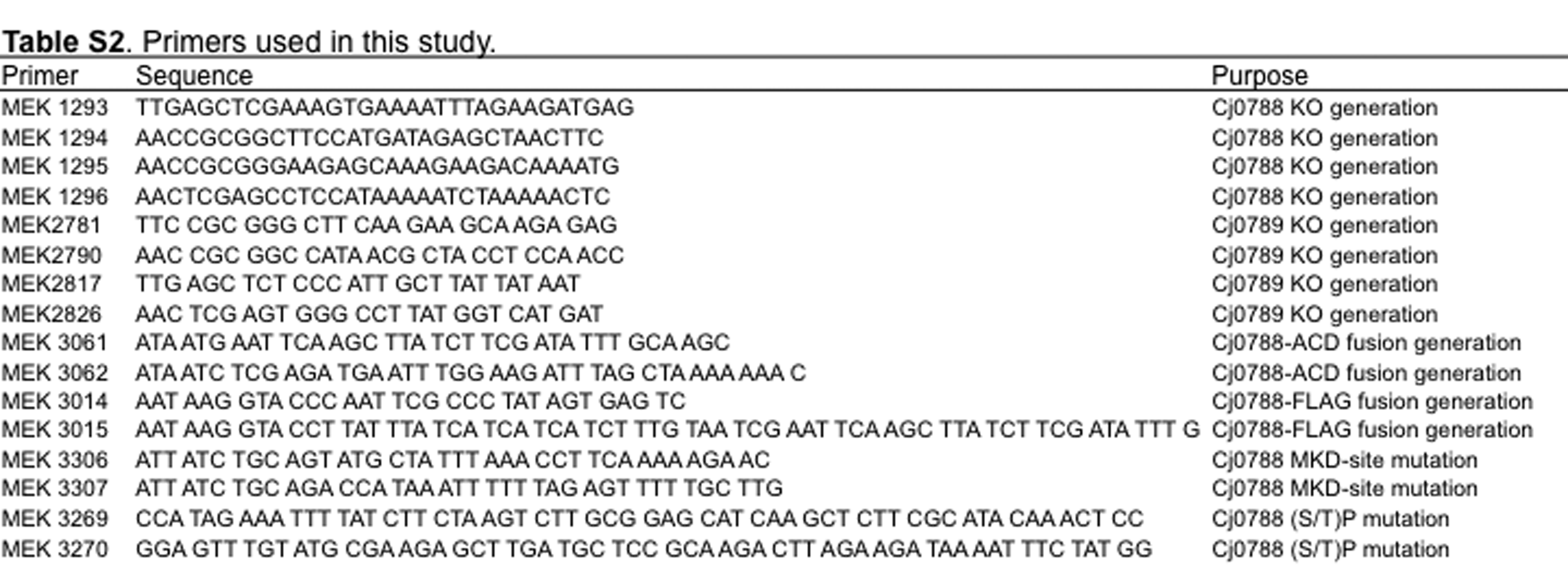

Supplement: Additional file 12: Table S2 — Primers used in this study. [file 1478-811X-11-79-S12.tiff]
